# Small sinking particles control anammox rates in the Peruvian oxygen minimum zone

**DOI:** 10.1038/s41467-021-23340-4

**Published:** 2021-05-28

**Authors:** Clarissa Karthäuser, Soeren Ahmerkamp, Hannah K. Marchant, Laura A. Bristow, Helena Hauss, Morten H. Iversen, Rainer Kiko, Joeran Maerz, Gaute Lavik, Marcel M. M. Kuypers

**Affiliations:** 1 Max Planck Institute for Marine Microbiology, Bremen, Germany; 2 MARUM—Center for Marine Environmental Sciences, University of Bremen, Bremen, Germany; 3 GEOMAR Helmholtz Center for Ocean Research Kiel, Kiel, Germany; 4 Alfred Wegener Institute Helmholtz Center for Polar and Marine Research, Bremerhaven, Germany; 5 Max Planck Institute for Meteorology, Hamburg, Germany; 6 Department of Biology, University of Southern Denmark, Odense, Denmark; 7 Laboratoire d’Océanographie de Villefranche-sur-Mer, Villefranche-sur-Mer, France

**Keywords:** Element cycles, Element cycles, Marine biology

## Abstract

Anaerobic oxidation of ammonium (anammox) in oxygen minimum zones (OMZs) is a major pathway of oceanic nitrogen loss. Ammonium released from sinking particles has been suggested to fuel this process. During cruises to the Peruvian OMZ in April–June 2017 we found that anammox rates are strongly correlated with the volume of small particles (128–512 µm), even though anammox bacteria were not directly associated with particles. This suggests that the relationship between anammox rates and particles is related to the ammonium released from particles by remineralization. To investigate this, ammonium release from particles was modelled and theoretical encounters of free-living anammox bacteria with ammonium in the particle boundary layer were calculated. These results indicated that small sinking particles could be responsible for ~75% of ammonium release in anoxic waters and that free-living anammox bacteria frequently encounter ammonium in the vicinity of smaller particles. This indicates a so far underestimated role of abundant, slow-sinking small particles in controlling oceanic nutrient budgets, and furthermore implies that observations of the volume of small particles could be used to estimate N-loss across large areas.

## Abstract (web summary)

Up to 40% of the ocean’s fixed nitrogen is lost in oxygen minimum zones (OMZs) by anammox, but despite the importance of this process, nitrogen loss patterns in OMZs are difficult to predict. Here the authors show that ammonium release from small particles is a major control of anammox in the Peruvian OMZ.

## Introduction

The upwelling of cold, nutrient-rich water drives high primary production rates in the surface waters of (sub)tropical eastern ocean boundaries. The resulting export of organic matter in the form of aggregates and particles fuels high microbial respiration rates which, combined with sluggish ventilation, leads to the formation of oxygen minimum zones (OMZs). OMZs (when defined by O_2_ ≤ 20 µmol L^−1^)^1^ make up <1% of the ocean volume, however 20–40% of global oceanic fixed nitrogen (N)-loss occurs within them^2–4^ due to the microbially mediated processes of anammox (anaerobic oxidation of ammonium^1,5–7^) and denitrification^8–10^. Hence, despite their small volume, OMZs have a profound impact on biogeochemical cycles.

In the past decades, OMZs have expanded substantially^11,12^, and climate models predict that global warming will accelerate deoxygenation^13^. However, the current and future extent of OMZs, and N-loss patterns within them, remain difficult to reproduce and predict in current Earth System Models^14–16^. To solve this issue, the drivers and impacts of physical and biological processes including ventilation, net primary production, respiration and carbon export need to be better represented in models^14,17–19^. For example, the representation of OMZs, as well as estimates of N-loss in low oxygen waters has been improved by the inclusion of aggregate and particle dynamics into biogeochemical models^20,21^. However, the role that sinking particles play in regulating N-loss within anoxic waters remains poorly understood.

It has been shown previously that rates of anammox correlate strongly with satellite-derived estimates of organic nitrogen export out of the photic zone by sinking aggregates and particles^5^. Generally, this correlation has been attributed to the release of ammonium associated with the remineralization of sinking organic matter^5,9^. Ammonium is a key substrate for anammox, but is present at very low concentrations in OMZ waters. Although anammox bacteria colonize resuspended organic particles within some OMZs^22^, it is currently unclear how the microorganisms that carry out anammox interact with sinking organic material and gain access to particle-derived ammonium. Whilst incubation experiments using size-fractionated water samples have indicated that anammox rates are higher in the presence of particles, metagenomic and metatranscriptomic studies have indicated that anammox bacteria reside in the water column, rather than on the particles themselves^23–26^.

The influence of differently sized particles is likely a key factor required to understand the mechanistic link between organic matter which sinks into anoxic OMZ waters, the apparently free-living anammox community and associated N-loss rates. In general, if composed of the same material, smaller particles sink slower than larger particles, thus smaller particles have longer residence times and are older by the time they reach deeper waters^27–29^. Furthermore, organic carbon appears to be respired more quickly on smaller and suspended particles^30,31^. Together these factors mean that smaller particles undergo more remineralization than larger particles as they sink through the water column^27^. Whilst this means that smaller particles have low transfer efficiencies in terms of carbon export from the subsurface to deep waters^27,32,33^, the remineralization of smaller particles has the potential to play an important role in releasing ammonium within the upper anoxic water column, where anammox rates are often highest^8,34^.

In this study, we aimed to constrain the relationship between anammox rates, the export of organic nitrogen out of the photic zone, and the abundance and total volume of sinking particles within the anoxic part of the Peruvian Upwelling System OMZ. The Peruvian Upwelling System forms part of the boundary current system of the Eastern Tropical South Pacific and is one of the most biologically productive regions in the world^35^. During austral autumn (April and June 2017), we carried out anammox rate measurements in anoxic bulk and size-fractionated waters and correlated them to imaged particle abundance and volume at high resolution over depth profiles. Thereafter, to gain mechanistic insights into the relationship between particles and anammox rates, we used the in situ particle profiles combined with derived settling velocities to model export of organic nitrogen by sinking particles, N-release associated with different particle size classes within the OMZ, and encounter rates of anammox bacteria with particles. We also used the measured anammox rates and particle abundances to derive a simple relationship in order to extrapolate anammox rates across the anoxic waters of the Peruvian OMZ at high spatial resolution.

## Results and discussion

### Physico-chemical conditions in the Peruvian Upwelling System

**Fig. 1:**
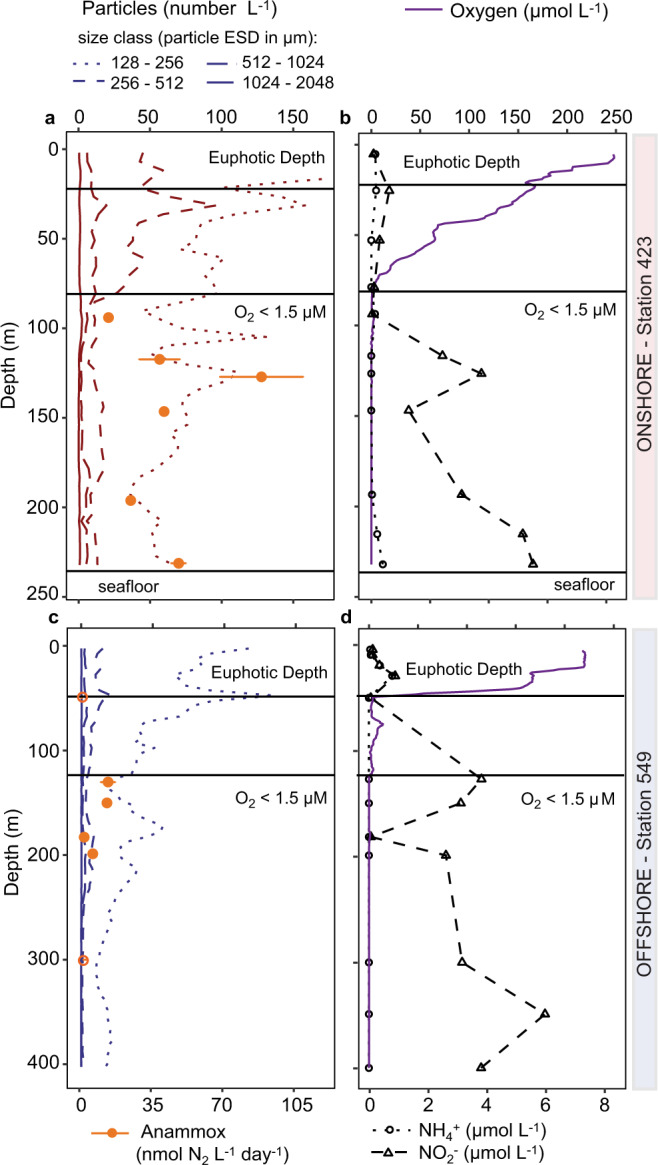
Physico-chemical parameters, particle abundance and anammox rates from an exemplary onshore and offshore station in the Peruvian upwelling system in April 2017. In situ particle abundances and anammox rates from **a** onshore station 423 (total water depth 241 m) and **c** offshore station 549 (total water depth 4350 m). **b** and **d** Depth profiles of oxygen (purple line), ammonium (open circles) and nitrite (open triangles) concentrations. Particles were sorted into four size classes with equivalent spherical diameters (ESD) of 128–256, 256–512, 512–1024, and 1024–2048 µm. Particle abundances in the smallest size class reached up to 398 particles L^−1^ in the upper 20 m of the onshore station. Anammox rates were determined in time series incubations at six discrete depths. Significant rates are shown with closed circles, rates that are not significantly different from zero are shown with open circles (see Supplementary Table 1). Error bars represent the standard error of the slope. The base of the euphotic zone (where photosynthetically active radiation (PAR) dropped to <1%, based on Aqua-MODIS satellite data) and the oxic–anoxic interface (where O_2_ dropped to <1.5 µM) are indicated.

The influence of sinking particles and aggregates on anammox rates was investigated on two cruises to the Peruvian Upwelling System in the Eastern Tropical South Pacific in austral autumn (April and June 2017). These cruises took place during the declining phase of a Coastal Warming Event, which was characterized by an unusual development of warm water off the coast of Peru while neutral sea surface temperatures persisted in the equatorial Pacific^36–39^. As a result, increased surface water temperatures of up to 24 °C were observed at some of the sampled stations (Supplementary Fig. 1). Despite this unusual event, most other conditions in the OMZ were typical for austral autumn^5,7,8,34,40,41^. The oxic–anoxic interface (throughout defined as where O_2_ dropped below 1.5 µM, which was the detection limit of the sensor) was generally around 50–100 m water depth (Fig. 1 and Supplementary Fig. 1), although, as is typical for this region, at some shallow onshore stations, the OMZ was observed to expand up to 10 m below the surface (Supplementary Fig. 1, Supplementary Tables 1 and 2). As observed previously, surface chlorophyll *a* concentrations were variable, both spatially and temporally (Supplementary Video 1, Supplementary Fig. 2c), nevertheless surface chlorophyll *a* was generally higher at the onshore stations (defined throughout as water depths <600 m), than at offshore stations (water depths >600 m). These depth cut-offs are based on ref. ^5^, where 600 m was the average depth of the lower OMZ boundary, roughly equivalent to the edge of the Peruvian shelf, and are henceforth used throughout to differentiate between onshore and offshore OMZ stations. Chlorophyll *a* concentrations were less variable over depth, and dropped below 0.2 mg L^−1^ by a depth of 50 m (Supplementary Fig. 1).

### Abundance and characterization of sinking organic material

**Fig. 2:**
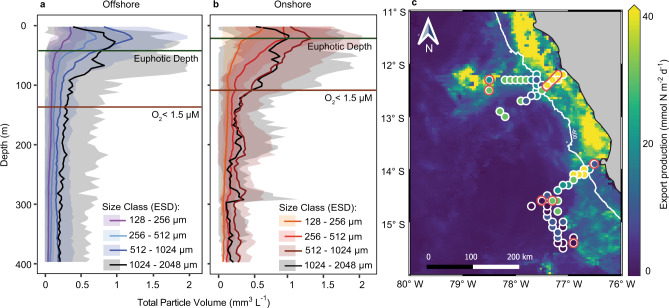
Particle volume and export production in the Peruvian upwelling system during April 2017. Average particle volume profiles in (**a**) offshore and (**b**) onshore stations determined by the Underwater Vision Profiler camera system (UVP). Particles were sorted into four size classes with equivalent spherical diameters (ESD) of 128–256, 256–512, 512–1024, and 1024–2048 µm. The shaded envelopes correspond to standard deviation in abundances from 56 offshore and 37 onshore stations (cropped at 2 mm^3^ L^−1^ for visualization purposes). The base of the euphotic zone (where photosynthetically active radiation (PAR) dropped to <1%, based on Aqua-MODIS satellite data) and the oxic–anoxic interface (where O_2_ dropped to <1.5 µM) are indicated. **c** Export production estimated from satellite products (pseudocolor map, see the “Methods” section) and from particle abundances at the base of the euphotic zone (circles). The white line is the 600 m isobath on which basis the onshore and offshore stations were separated. Stations where anammox incubations were performed are circled in red, the elongated red circle encompasses four stations.

To characterize the export of the particulate organic matter formed in the OMZ surface waters, the size and abundance of particles and aggregates with equivalent spherical diameters (ESD) between 128 and 2048 µm were determined from 5 m below the surface down to the seafloor at onshore stations and down to at least 500 m at offshore stations using an Underwater Vision Profiler (UVP; an underwater camera system that can be mounted to a rosette sampler^42^). Particles were subsequently binned into four size classes based on ESD (128–256, 256–512, 512–1024, 1024–2048 µm). The size classes were chosen to ensure that there was a similar total volume of particles within each size class, as particle abundance and volume generally decrease with increasing size^43,44^ (Supplementary Fig. 3). The average particle and aggregate volume was higher for the onshore stations than for the offshore stations at all depths (Fig. 2a and b). The total volume of the imaged particles generally peaked at the base of the euphotic zone at the onshore stations (up to 3.6 ± 2.8 mm^3^ L^−1^) and towards the centre of the euphotic zone at the offshore stations (up to 3.2 ± 2.4 mm^3^ L^−1^), after which they declined to minima of 0.5 mm^3^ L^−1^ in both the onshore and offshore OMZ (Fig. 2, Supplementary Figs. 1 and 2a and b). At some shallow onshore stations, particle and aggregate volume increased again towards the seafloor, likely due to sediment resuspension.

We assume that all of the imaged aggregates and particles were sinking, similar to previous studies based on in situ particle profiling with a UVP system^45,46^. While suspended particles may comprise a substantial amount of total POC in the ocean^27,30,47^, such particles are likely smaller than those imaged by the UVP (which were >128 µm). For example, marine microgels are generally <100 µm (ref. ^48^) and a recent study which examined in situ settling velocities with a camera system, observed that more than 95% of particles with ESDs larger than 80 µm were sinking with velocities >10 m day^−1^ in the mesopelagic zone^49^. Our assumption that the UVP imaged particles were sinking is also supported by the presence of particles in the 128–2048 µm size range within the tray of a Marine Snow Catcher, which is designed to only collect sinking particles.

Categorization of 910 particles and aggregates collected using a Marine Snow Catcher revealed that they were generally detrital material that appeared to be composed of similar matter independent of size class or packing density. Although the matter consisted of aggregated material rather than single particles, we hereafter refer to particles for simplicity. Particles in the two smaller size classes (128–256 and 256–512 µm) consisted mainly of loosely and densely packed detrital material (fluffy detritus, 44% and compact detritus, 34%), as well as some faecal pellets (6%) and various other types of particles such as dead zooplankton, foraminifera and other material that could not clearly be identified (15% other material). The two larger size classes (512–1024, 1024–2048 µm) consisted of loosely packed detrital material (fluffy detritus, 51%), loosely packed detrital material that contained faecal pellets (25%), densely packed detrital material (compact detritus, 11%) and other material (11%). Only a small proportion of the larger size classes consisted of individual faecal pellets (3%, Supplementary Table 3). Overall, these categorizations were consistent with those from in situ observations made using the UVP and are representative of the sinking, but not the suspended material. The UVP profiles indicated that particles within the smallest size class (128–256 µm) were the most abundant at all depths (Supplementary Table 1) and overall abundance decreased with increasing particle size. The observed distribution of particles over depth and between size classes is consistent with studies in other ocean regions, where a higher export of smaller particles leads to their comparatively greater abundance below the euphotic zone^45,47,50,51^. Additionally, the higher abundance of smaller particles in the OMZ core might result from the break-up of larger aggregates through fragmentation (Supplementary Discussion)^20,45,47,50,51^ or active transport by migrating zooplankton^52,53^, although this latter option is unlikely as few faecal pellets were observed at this depth.

### Export of particles as a source of N for anammox in the Peruvian OMZ

To determine the links between sinking organic matter and N-loss rates in the Peruvian OMZ, we determined anammox and denitrification rates in bulk water collected from the anoxic region of the OMZ and compared them to particle abundances and volume. On average, anammox rates were 33.5 ± 26.1 nmol N_2_ L^−1^ day^−1^ at onshore stations and 8.3 ± 15.4 at offshore stations (Table 1, Supplementary Table 1, Supplementary Fig. 1). Rates were generally highest 20–60 m below the oxic–anoxic interface after which they decreased with increasing depth. Both the offshore rates and comparatively high rates at the productive onshore stations (which reached up to 89.6 nmol N_2_ L^−1^ day^−1^), as well as the rate distribution over depth are consistent with those measured before in the Eastern Tropical South Pacific^5,7,8,34,40,41,54–57^. In comparison, denitrification rates were on average 13.5 and 4.5 times lower than anammox rates at onshore and offshore stations, respectively (Table 1, Supplementary Table 1 and Supplementary Fig. 4). Based on these results, denitrification was a minor N-loss process in the Peruvian OMZ during austral autumn, similar to previous observations^5,54^.

**Table 1:** Averaged and integrated anammox and denitrification rates, and export of organic nitrogen out of the euphotic zone into the oxygen minimum zone (OMZ) from all sampled onshore and offshore stations (for more information refer to Supplementary Tables 1a and 2).

		Unit	Onshore (mean ± SD)	Offshore (mean ± SD)	Combined (mean ± SD)
OMZ average^a^	Anammox	nmol N_2_ L^−1^ day^−1^	33.5 ± 26.1	8.3 ± 15.4	20.1 ± 24.4
	Denitrification		2.5 ± 4.0	1.8 ± 2.4	2.1 ± 3.2
Integrated rate over depth^a^	Anammox	mmol N_2_ m^−2^ day^−1^	3.1 ± 3.3	1.2 ± 1.7	2.2 ± 2.8
	Denitrification		0.2 ± 0.5	0.5 ± 0.5	0.4 ± 0.5
Organic nitrogen export	128–256 µm	mmol N m^−2^ day^−1^	8.0 ± 7.9	6.0 ± 2.1	7.1 ± 5.8
	256–512 µm		6.7 ± 5.1	4.5 ± 0.9	5.7 ± 3.8
	512–1024 µm		5.3 ± 4.5	3.2 ± 1.5	4.4 ± 3.5
	1024–2048 µm		2.1 ± 1.9	1.2 ± 1.0	1.7 ± 1.5

^a^In situ oxygen <1.5 µM.

The export of organic nitrogen out of the photic zone was calculated using two methods. One method was based on satellite measurements of chlorophyll *a* and temperature and an assumed particle export to primary production ratio (similar to ref. ^5^). The other method was based on combining the in situ measurements of particle abundance and size (using the UVP camera system) with measured particle carbon contents, previously determined C:N ratios and calculated settling velocities (UVP method). Both methods resulted in similar estimates of organic nitrogen export, although those from the satellite were slightly lower (Supplementary Discussion and Supplementary Fig. 5). As the UVP-based estimates were carried out simultaneously to the water sampling, they capture the local variability of organic nitrogen export more precisely, and therefore we focus on the UVP dataset for comparison to the anammox rates. Based on the UVP method, organic nitrogen export from the photic zone ranged between 6.5 and 61.7 mmol N m^−2^ day^−1^ onshore, and 6.9 and 45.7 mmol N m^−2^ day^−1^ offshore (Fig. 2c, Supplementary Fig. 2d), and was always at least 1.8-fold higher than depth-integrated anammox rates (Supplementary Table 2 and Supplementary Fig. 6). As such, the export of organic nitrogen was sufficient to support the measured N-loss, which is in accordance with previous results from the Peruvian OMZ^5^.

### Anammox bacteria are free-living

To further investigate the relationship between anammox rates and export of organic N via sinking particles, we determined where anammox activity was located within the anoxic waters of the OMZ. Previous studies based on size-fractionated water samples have indicated that anammox bacteria are more likely to be part of the free-living pelagic community than the particle-associated community in various OMZs, including the Eastern Tropical North Pacific, Eastern Tropical South Pacific, Black Sea and Cariaco Basin^23–25,58^. However, FISH-based studies have indicated that anammox bacteria can be associated with particles in the Namibian OMZ where sediment resuspension is high^22^.

**Fig. 3:**
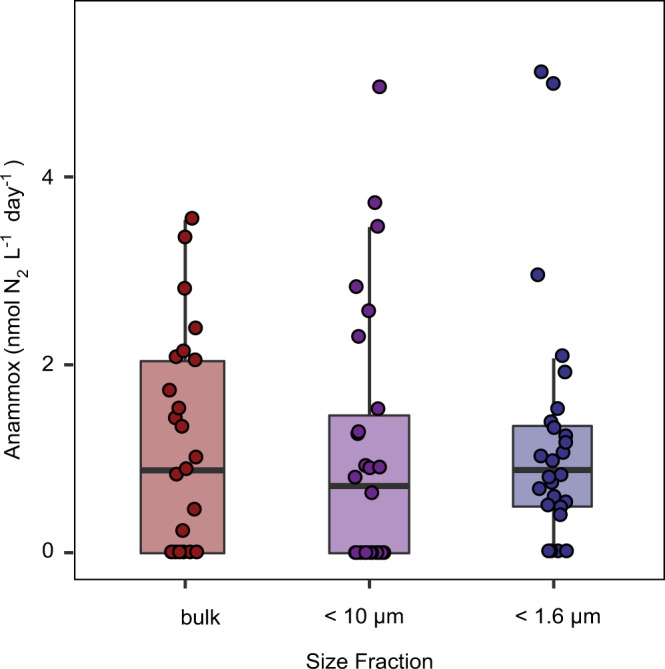
Anammox rates determined from unfiltered water (bulk) and water filtered through either a 10 or 1.6 µm filter in the anoxic waters of the Peruvian upwelling system during April and June 2017. Boxplots depict the 25–75% quantile range, with the centre line depicting the median (50% quantile); whiskers encompass data points within 1.5 times the interquartile range. A paired *t*-test (Supplementary Table 4) showed that there was no significant difference in rates between any of the treatments. Anammox rates from one depth are outside the range of the graph and had anammox rates of 14.1 nmol N_2_ L^−1^ day^−1^ (bulk water), 41.1 nmol N_2_ L^−1^ day^−1^ (10 µm filtered) and 8.7 nmol N_2_ L^−1^ day^−1^ (1.6 µm filtered). These values were included in the statistical analysis. At an additional 23 depths anammox rates were below detection limit in all size fractions and these were not included in the analysis. See Supplementary Fig. 7 for individual data points and Supplementary Table 1b for the standard error of the slope of each individual rate measurement.

The bulk water incubations that we carried out already indicated that anammox activity was unlikely to be occurring directly on particles (see Supplementary Discussion). To confirm that anammox activity was not directly associated with the particles, we carried out size-fractionation experiments at 11 stations to compare anammox rates in water that was either unfiltered, or in water where particles had been removed by filtration through a 1.6 µm filter or a 10 µm filter. Anammox rates in the <10 µm fraction, the <1.6 µm fraction and bulk water samples (i.e. non-fractionated) were not significantly different from each other (Fig. 3, Supplementary Fig. 7 and Supplementary Table 4). This indicates that anammox bacteria were not removed along with the particles by the filtration steps, and are therefore most likely free-living within the anoxic OMZ waters rather than attached to particles.

### Correlation of anammox rates to total particle volume

**Fig. 4:**
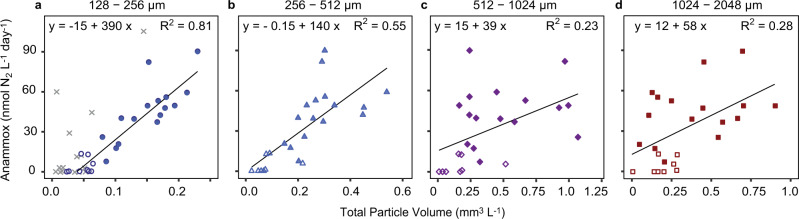
Relationship between volumetric anammox rates and particle volumes from different size classes in the Peruvian upwelling system during April 2017. Particles throughout the water column were quantified with an Underwater Vision Profiler camera system (UVP) and binned into four size classes with equivalent spherical diameters (ESD) of 128–256, 256–512, 512–1024, and 1024–2048 µm. Anammox rates were determined from the slope of ^29^N_2_ production over time in anoxic incubations after addition of ^15^NO_2_^−^ (taking into consideration any contribution to ^29^N_2_ production from denitrification if a denitrification rate with *p* < 0.05 could be detected). For all particle size classes, there was a significant positive correlation (Spearman’s rank correlation; *p* = <0.05) between particle volume and anammox rates from the anoxic part of the oxygen minimum zone (O_2_ < 1.5 µM). The line is the linear regression from which the slope and *R*^2^ was calculated (see Supplementary Table 5 for all relevant statistics). Please note the different scales for the particle volume **a**–**d**. One outlier was removed from the figures and correlation as it was more than 6 standard deviations from the mean in the smallest size fraction and it was the only sample from the euphotic zone (24.3 m depth). In **a**–**d**, open and filled symbols depict offshore stations and onshore stations, respectively. In panel **a**, the correlation with the smallest size class (140–270 µm) during a cruise in February 2013 (M93) is included for comparison and depicted with grey x symbols. This dataset was not included in the linear regression but is included in Supplementary Table 1c. Correlations with the particle abundance in number per liter yielded similar results (see Supplementary Table 5).

To constrain the relationship between the apparently free-living anammox bacteria and particles within the OMZ, anammox rates from the bulk water incubations were compared to the total volume of particles within different size classes determined at the same time and depth with the UVP (Fig. 4). We found significant positive correlations between anammox rates from anoxic waters and the corresponding volume of particles within each of the particle size classes (Spearman’s rank correlation; *p* < 0.05, see Fig. 4, Supplementary Fig. 8 and Supplementary Table 5). These correlations were strongest for the two smaller size classes. The observed stronger correlation between anammox rates and smaller particles was further supported by a multiple linear regression analysis, which revealed that the volume of the smallest particles was the most important variable needed to predict anammox rates (see Supplementary Table 6 for more details). The slope of the correlation between anammox rates and particles in the smallest size class (128–256 µm) was 390 nmol N_2_ mm^−3^ day^−1^.

Intriguingly, we also found a significant linear correlation between anammox rates and the volume of the smallest particle size class, during a previous cruise (M93) to the same region in February 2013 (Fig. 4a, Supplementary Fig. 9), although the slope was higher (~590). It should be noted that in contrast to the dataset presented here, the M93 cruise occurred in austral summer when productivity is relatively high and the water column was sulfidic at some stations. We excluded the data from those stations, as sulfidic events have been shown to alter N-loss patterns in the Peruvian OMZ^34,40,59,60^. Nonetheless, the similarity in the correlation indicates that the positive relationship between measured anammox rates and measured volume of smaller particles is a consistent feature within the Peruvian OMZ. To explore the underlying mechanisms of this relationship, we compared the organic N-export flux associated with each particle size class, modelled the potential ammonium release by the particles and estimated the likelihood of anammox bacteria to encounter the released ammonium.

### Modelling of potential nitrogen release from different particle size classes

**Fig. 5:**
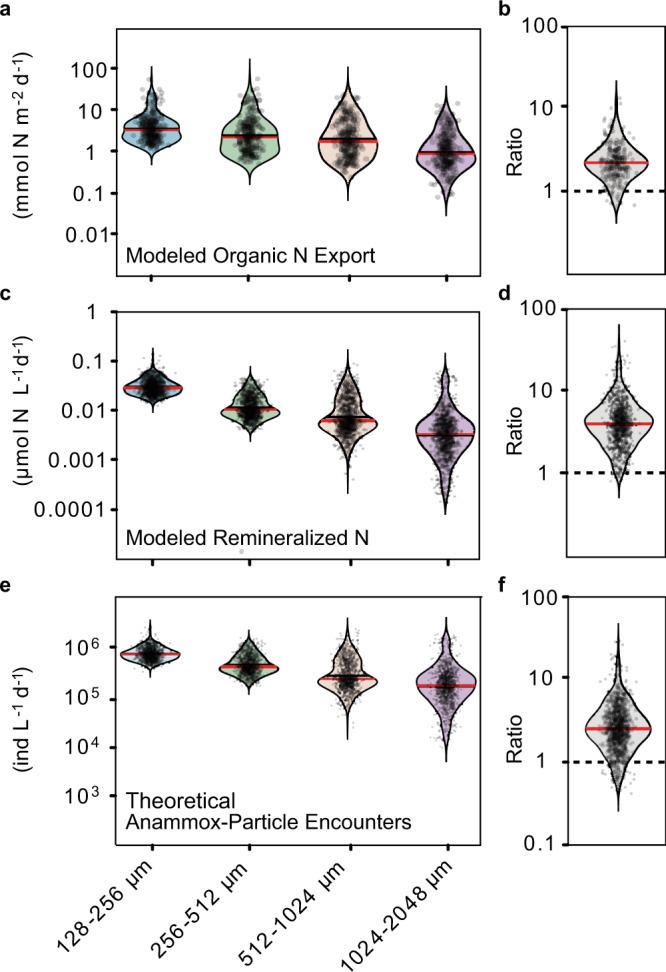
Transport of nitrogen (N) from the euphotic zone through the water column. **a** Modelled export of organic nitrogen from the euphotic zone into the oxygen minimum zone, **c** modelled nitrogen remineralization associated with particles in the anoxic water column (**e**) theoretical number of encounters between anammox bacteria and the particle boundary layer (see text for further information). **b**, **d** and **f** indicate the ratio of processes between the smaller (128–512 µm) and larger particles (512–2048 µm) from (**a**, **c** and **e**,) respectively. Ind: individuals. The outlines of the violin plots depict the kernel density estimation of the data points shown. The solid black line and solid red line indicate mean and median, respectively.

The export of organic nitrogen into the anoxic waters of the OMZ was modelled for each particle size class based on measured particle abundance at the base of the euphotic zone, measured size-specific carbon content, calculated sinking velocities and a C:N value of 7.2 as was determined previously for the region (Eq. (1)). The model assumes that all of the imaged particles were sinking (see above). Overall, when all the stations where the UVP was deployed were taken together, the smaller particles (<512 µm) were estimated to be responsible for the export of 1.8 times more organic nitrogen into the anoxic zone than the larger size classes (Fig. 5a, b). This relationship between the size classes persisted when a different function was used to calculate sinking velocities (Supplementary Fig. 10) and when using size-dependent C:N ratios (Supplementary Fig. 11 and Supplementary Discussion) to represent the preferential remineralization of organic N that often occurs during organic matter degradation (see Supplementary Discussion and Supplementary Figs. 9–13)^28,29,61–63^.

The amount of organic nitrogen bound in particles that enter the OMZ is only the first step in substrate supply to anammox bacteria. The particulate organic nitrogen must subsequently be remineralized to inorganic nitrogen (i.e. ammonium). The continual decline in total particle numbers and volume with depth implied that remineralization of organic carbon and nitrogen within particles occurred throughout the OMZ. The highest carbon flux attenuation was observed within the upper oxic waters of the OMZ, indicating that this was where remineralization rates were highest (Supplementary Fig. 15). In this zone, carbon-specific degradation rates were estimated to be between 0.33 and 1.6 day^−1^ (see Supplementary Discussion), which is in line with previous rates from the Eastern Tropical North Pacific determined from measurements of oxygen respiration (0.13–5 day^−1^, ref. ^30^). Based on a median particle number of 66 particles per liter this is equivalent to remineralization of between 3.2 and 6.0 µmol C L^−1^ day^−1^ (Supplementary Table 8), or, assuming a C:N ratio of 7.2, N-remineralization of between 0.44 and 0.83 µmol N L^−1^ day^−1^.

By comparing the time scale of diffusion with the settling velocity, we also estimated the distance that particles could sink before remineralized nitrogen would mostly diffuse into the water column. This so-called diffusion length scale ranged between 0.2 mm for the smallest particles and 20 cm for the largest particles. These results indicate that reduced nitrogen formed by remineralization in the oxic, euphotic water column mainly diffuses out of the particles within that same zone. It is therefore unlikely that particles transport ammonium generated by aerobic remineralization in the upper water column into the anoxic zone. Therefore, while the diffusive release of reduced nitrogen is likely a key factor that drives the enhanced N-cycling activity that is frequently observed at the oxic–anoxic interface within OMZs^5,7,41,55,64^, it would not support anammox activity below the oxic–anoxic interface.

Anammox activity within the rest of the OMZ therefore seems to be supported by anaerobic remineralization and subsequent nitrogen release from particles within the anoxic part of the OMZ. Carbon-specific degradation rates (derived from the UVP measurements and measured particle carbon content, see Supplementary Fig. 16) from 50 m below the oxic–anoxic interface were estimated to be on average 0.22 day^−1^ onshore and 0.15 day^−1^ offshore. This would result in a remineralization of 0.3 µmol C L^−1^ day^−1^ at onshore stations and 0.16 µmol C L^−1^ day^−1^ at offshore stations. Assuming a C:N stoichiometry of 7.2 for the sinking organic matter (see ref. ^5^), this would be equivalent to the release of 0.04 and 0.02 µmol N L^−1^ day^−1^. Of this, 71% and 76%, (on average 74%) could be attributed to the smaller particle size classes at the onshore and offshore stations, respectively (Eq. (7), Fig. 5c, d). The relatively higher importance of smaller particles persisted even when varying the parametrization of the settling velocity and C:N ratios in the model and when considering the impact of particle fragmentation (see Supplementary Discussion).

Intriguingly, the modelled estimates of nitrogen release from the particles were higher than the organic matter remineralization (i.e. ammonium release) that is estimated to occur in the Peruvian OMZ via heterotrophic nitrate-based and nitrite-based respiratory processes (i.e nitrate reduction to nitrite, denitrification and dissimilatory nitrate/nitrite reduction to ammonium, DNRA). Nitrate reduction to nitrite, which is suggested to be the main remineralization process, has typical onshore rates of 0.2 µmol N L^−1^ day^−1^ (refs. ^5,55^). Remineralization associated with nitrate reduction to nitrite would therefore lead to the release of around 0.015 µmol NH_4_^+^ L^−1^ day^−1^, around 40% of our estimated ammonium release (0.04 µmol N L^−1^ day^−1^), which is likely underestimated. Preferential N-mineralization within particles and particle fragmentation (which cannot be fully resolved via modelling with this dataset) could both theoretically lead to higher ammonium release (see Supplementary Discussion). Furthermore, our modelling approach only takes sinking particles into account, whereas it is possible that some fraction of the smaller particles are suspended in the water column^27^. If this were the case, we would underestimate N-release, as suspended particles would have a longer residence time in the OMZ (see Supplementary Discussion). Our results therefore imply that non-nitrate-based and nitrite-based respiratory processes lead to organic nitrogen remineralization within the particles. Such processes might be microaerobic respiration by prokaryotes^64–66^, and microzooplankton transported within particles, or fermentation, which has recently been shown to be a significant source of ammonium within the Eastern Tropical South Pacific OMZ^67^.

### Encounters between anammox bacteria and remineralized nitrogen released by particles

Remineralized organic nitrogen released from sinking particles does not immediately diffuse evenly throughout the water column. Instead, ammonium and other solutes diffuse from particles into their boundary layer, leaving a plume in the wake of sinking particles (see Supplementary Fig. 12, refs. ^68–70^). The concentrations of nutrients in the diffusive boundary layer and plume tails can be two to three orders of magnitude higher than in the surrounding seawater^70–73^. To estimate encounter rates between free-living anammox bacteria and the diffusive boundary layer and plumes of particles, we combined particle abundance data with sinking velocities, boundary layer theory (Eq. (8), Fig. 5e) and previously determined anammox bacterial cell numbers^41^. The resulting encounter model showed an average of 125 encounters per hour per mL (regardless of size class), which equates to 0.3% of the anammox bacteria population encountering a particle-associated nutrient hotspot every hour. When combined with typical cell-specific anammox rates of ~1–10 fmol cell^−1^ h^−1^ (refs. ^74,75^), these encounter rates would support in situ rates between 3 and 30 nmol L^−1^ day^−1^, which is in line with the measured rates (Table 1). It is likely that this is a conservative estimate as the encounter model assumes that anammox bacteria are not motile^76,77^, which limits encounters with sinking particles and resulting nutrient hotspots. So far, *Scalindua* (which are the most abundant anammox bacteria within the Peruvian OMZ^78^) are not known to be motile, however it should be noted that for motile bacteria at the same cell numbers, encounter rates could be increased by 179–400 times (Supplementary Fig. 14; Supplementary Discussion)^79^.

The encounter model revealed that on average, non-motile anammox bacteria were between 1.5 and 2.6 times more likely to encounter the ammonium diffusing from particles within the two smaller size classes than that from the larger size classes (Fig. 5f, Supplementary Fig. 10). This was mainly due to the higher abundance and increased boundary layer thickness to size ratio of the smaller particles (Supplementary Fig. 12; Supplementary Discussion). For motile bacteria, this estimate would increase to 3.8 times. This further highlights the important role that smaller particles seem to play in regulating anammox rates and regional nitrogen loss.

### Extrapolation of anammox to regional scales based on particle abundances

**Fig. 6:**
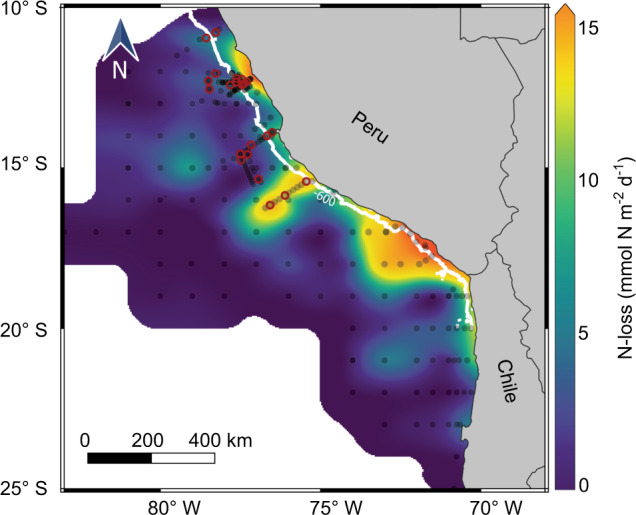
Regional estimate of nitrogen (N)-loss via anammox based on the volume of small particles (128–256 µm). Anammox rates in the anoxic water column of Peruvian oxygen minimum zone (O_2_ < 1.5 µM) derived from UVP measurements carried out from March to July 2017 (black circles depict UVP casts during cruises M135–M138). Extrapolating these rates over a whole year would lead to a nitrogen loss of around 19 Tg. The red circles depict stations where the anammox rates were measured. The white line is the 600 m isobath.

The simple linear relationship between the measured volume of small (128–256 µm) particles and measured anammox rates within the anoxic part of the OMZ (Fig. 4a) suggests that the distribution of small particles can be used to estimate nitrogen loss rates over large areas. The slope of the linear regression between particle volume in the smallest size class (128–256 µm) and anammox rates (390・*V*_126-256_−15; Fig. 4a), implies an N_2_ production per particle volume of 390 nmol N_2_ mm^−3^ day^−1^. Furthermore, the negative intercept implies that a minimum volume of small particles (0.04 mm^3^ L^−1^) is required to fuel a significant anammox rate. This is supported by the observation that anammox is undetectable in the OMZ waters containing very few particles. Based on our mechanistic modelling, the cessation in anammox rates at low particle volumes likely results from ammonium release and encounter rates becoming too low (see Supplementary Fig. 17). The relationship from the linear regression was applied to a UVP dataset which covered a large area of the Peruvian OMZ and was collected during March–July 2017 (cruises M135–M138). UVP measurements were separated for the “inner” and “outer” OMZ, based on distance from the coast (175 km, as in refs. ^5,41,80^), which led to an estimated average nitrogen loss within the anoxic waters of around 6.1 and 1.5 mmol N m^2^ day^−1^ for the inner and outer OMZ, respectively (Fig. 6). Subsequently, we extrapolated these rates over the area of the inner Peruvian OMZ (3.2 × 10^5^ km^2^, as in refs. ^41,80^) and the outer Peruvian OMZ (12 × 10^5^ km^2^ as in ref. ^5^). This resulted in an estimated monthly N-loss of 1.0 Tg N, and 0.6 Tg N for the inner and outer OMZ, respectively, during the period covered by this dataset.

If we extrapolate the monthly rates across an entire year, they would lead to an N-loss estimate of 19 Tg N yr^−1^ for the anoxic part of the Peruvian OMZ. This annual N-loss estimate is very similar to previous estimates which range from 10 to 25 Tg N yr^−1^ (refs. ^5,41,80–82^). This annual extrapolation would be improved by additional data from the early winter to early summer seasons, sulfidic events and also by the inclusion of anammox activity from the oxycline, which we did not investigate in this study. However, it demonstrates the potential of using particle size distribution and abundances to extrapolate measured anammox rates. Quantification of nitrogen loss in OMZs is often based on a few measurements extrapolated to a large region, potentially leading to uncertainties related to spatial heterogeneities. In contrast, profiling of aggregate and particle abundance is becoming a standard oceanographic tool, with camera systems such as the UVP often installed on rosette-CTD samplers^83,84^. Therefore, the application of this approach in combination with rate measurements in other OMZs and seasons would allow data to be collected at a high resolution over large areas, which can then be integrated into Earth System Models.

**Fig. 7:**
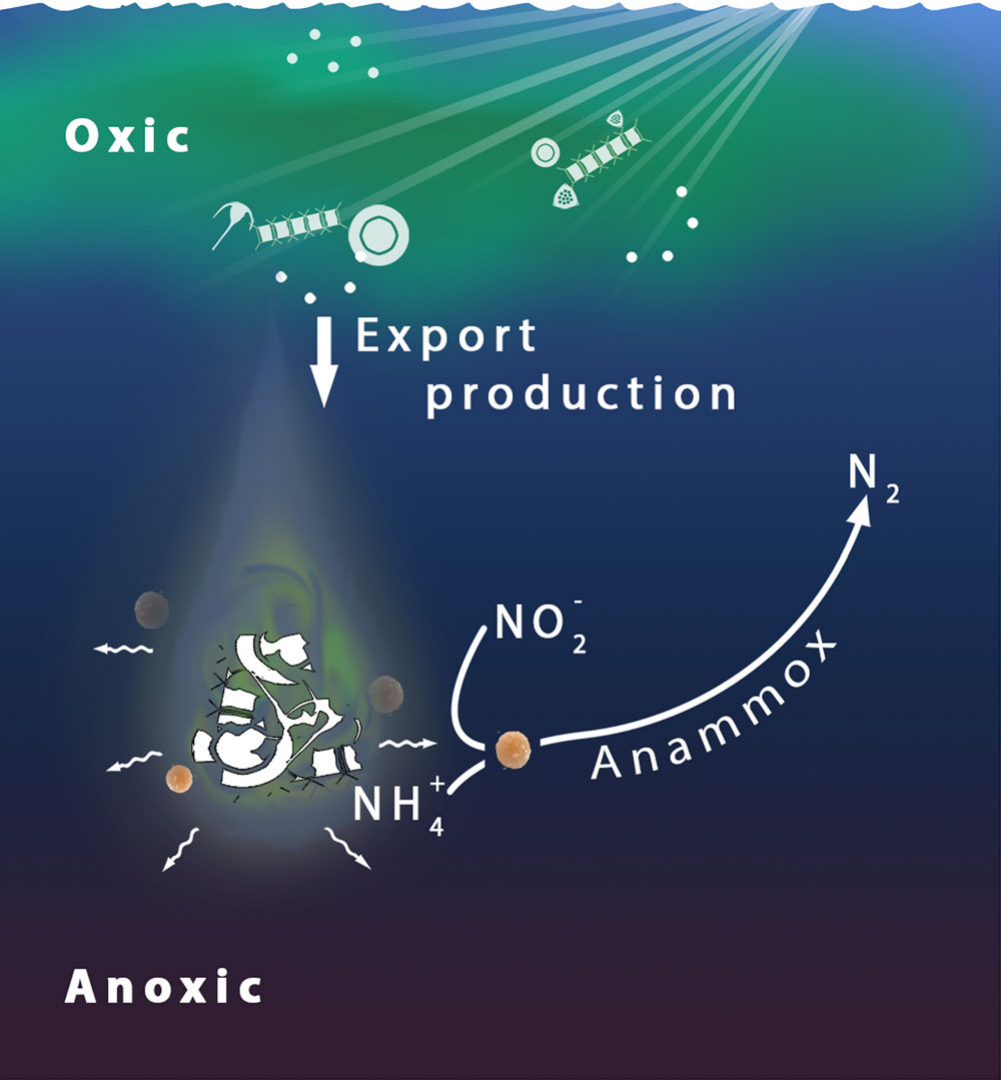
Schematic illustration showing interactions between anammox bacteria and particles within the Peruvian oxygen minimum zone (OMZ). Aggregated organic matter formed by primary production in euphotic surface waters sinks into the anoxic OMZ (export production). Subsequently, remineralization of the particles leads to the formation of ammonium, the limiting substrate for anammox. The ammonium diffuses into the particle boundary layer, leaving a plume in the wake of the sinking particles. Free-living anammox bacteria encounter the ammonium and utilize it as a substrate to carry out anammox, leading to the formation of dinitrogen gas (N_2_).

Our results show that anammox rates in the OMZ of the Peruvian Upwelling System are correlated to the abundance and volume of sinking particles. Furthermore, we show that highly abundant smaller, slower sinking particles may play a greater role in controlling anammox rates than rarer, faster sinking larger particles. Modelling approaches showed that the more abundant smaller particles transport between 1.2 and 1.8-fold more organic nitrogen into the OMZ than larger particles. Smaller particles also release around 75% more remineralized nitrogen within the OMZ than larger particles, due to their higher abundance and slower sinking rates. Furthermore, anammox bacteria are twice as likely to encounter remineralized nitrogen released from smaller particles (Fig. 7). Generally, smaller sinking particles are considered to have less of an impact on biogeochemical cycles than larger particles, as they have a lower carbon transfer efficiency from the upper water column to the deep sea. However, our results highlight an important role of smaller, slower sinking particles in controlling nitrogen loss, and show that their abundance and distribution could be used to estimate nitrogen loss across large areas. Taking the role of smaller particles into account in Earth System Models may have the potential to more accurately predict global nitrogen loss patterns in the present and future ocean.

## Methods

### Water sampling and physico-chemical measurements

A conductivity–temperature–depth (CTD)—rosette equipped with 24, 10 L Niskin bottles was used to collect water samples. Duplicate sensors were employed to record temperature and salinity (CTD type SBE 9-plus), oxygen (SBE 43 type oxygen sensor, Seabird) and fluorescence (WET Labs Fluorometer, USA). The precision for the salinity sensor was 0.002 g kg^−1^ and for the temperature sensor 0.002 °C. The detection limit of the oxygen sensor was 1.5 μmol L^−1^ (ref. ^85^) and it was calibrated regularly during the cruises against Winkler titration^86^. Chlorophyll *a* concentrations were derived from the fluorescence using the factory calibration^87^. High resolution in situ particle size spectra were determined using an UVP with an average pixel size of 151 µm (UVP5, ref. ^42^), which was attached to the CTD rosette. Particle sizes in the range 128–151 µm were determined using the well-established and standard regression approach^42^, therefore we were able to detect particles with ESD between 128 and 4096 µm (the upper and lower limits of the UVP system). The lowering speed of the CTD ranged between 0.5 and 1 m s^−1^ and data were averaged in 5 m depth bins. Particles within each depth bin were binned into size classes of 128–256, 256–512, 512–1024, 1024–2048 and 2048–4096 µm based on the pixel size of the UVP and to account for the decrease in particle abundance with increasing size. Particles in the 2048–4096 µm size bin were incredibly rare (on average 0.022 L^−1^) so they were excluded from further analysis.

Nutrient samples were collected during the CTD rosette upcast and measured immediately on board. Nitrate and nitrite measurements were carried out spectrophotometrically on a QuAAtro autoanalyzer (Seal Analytical GmbH, Germany) with a precision of 0.1 µmol L^−1^ (using a standard method as described by Hydes et al.^88^). Ammonium concentrations were measured according to the standard fluorometric method^89^.

### Anammox and denitrification rate measurements and depth-integration

Bulk incubation experiments to determine anammox rates were conducted at 13 stations, with 6 depths per station in most cases, which were distributed between the upper boundary of the OMZ (O_2_ < 10 µmol L^−1^) down to a depth of 464 m in the OMZ core (Supplementary Tables 1 and 2). For each incubation, water was filled into 250 mL serum bottles from the Niskin bottles using gas tight iso-versinic tubing. Bottles were allowed to gently overflow two to three times their own volume and then stoppered bubble-free with 20 mm butyl rubber septa (SUPELCO) that had previously been stored under a helium atmosphere. Subsequently ^15^N-labelled incubations were carried out as described by Dalsgaard et al. ^90^. Briefly, serum bottles were bubbled with helium for 15 min and amended with 5 µM ^15^NO_2_ after 10 min, after which they were bubbled with helium for another 5 min. Water was aliquoted through gas tight tubing into 12 mL glass vials (Exetainers, Labco Ltd), which were allowed to gently overflow with twice their volume before being capped headspace free. Caps were stored under a helium atmosphere for several months prior to use to reduce oxygen contamination^91^. Helium headspaces of 2 mL were introduced into each exetainer and flushed twice with helium^65^. Oxygen was monitored in a subset of the incubations, three glass vials per station were prepared with an optode spot (Pyroscience, TROXSP5 Trace Oxygen Sensor Spot) and filled at random during aliquoting, in addition to the incubation vials. Oxygen concentrations were measured using a fibre-optic cable connected to an amplifier (Pyroscience, Firesting, FSO2-4). The oxygen concentrations in the samples were always below the detection limit (0.06 µmol L^−1^). Exetainer incubation experiments were incubated in the dark at 14 ± 0.5 °C, which reflected the in situ temperature. Incubations were terminated after approximately 0, 6, 12 18 and 24 h by addition of 100 µL of a saturated mercuric chloride solution.

At 11 additional stations (7 offshore, 4 onshore; see Supplementary Table 1b) anammox rates were determined in size-fractionated samples. Water was collected from the CTD—rosette into 2 L glass bottles through gas tight tubing and allowed to overflow to avoid oxygen contamination. The bottle was sealed bubble free using a deoxygenated butyl rubber septa that had previously been stored under a helium atmosphere, using a needle to avoid the formation of a bubble. Subsequently, the water was degassed with helium for 15 min. The water was dispensed into 160 mL glass serum bottles using a helium overpressure through an inline 47 mm diameter filter holder (PALL, USA) which was flushed with helium to avoid oxygen contamination. For bulk water samples, no filter was added to the holder, for a second serum bottle, the filter holder contained a 10 µm pore size nylon filter (Millipore, Bellerica, MA, USA), and for the third set a 1.6 µm pore size filter was used (GF/A filter, Whatman, GE Healthcare Biosciences, Pittsburgh, PA, USA). Serum bottles were bubbled with helium for 10 min and the tracer was added (5 µM ^15^NO_2_) and then bubbled for a further 5 min before being dispensed into 12 mL glass vials (Exetainers, LabCo Ltd.), as described above. As a precaution against external microbial contamination, all bottles and hoses were washed with HCl before use, and sterilized stoppers and needles were used for each experiment. The aliquoted samples for the size fractionation incubations were then treated the same as the incubations described above.

Isotopic ratios of ^14^N^14^N, ^14^N^15^N and ^15^N^15^N dinitrogen gas were measured in the headspace of the incubation experiments with a GC–IRMS upon return to the laboratory in Bremen, Germany (customized TraceGas coupled to a multicollector IsoPrime100, Manchester, UK). The ^15^NO_2_^−^ labelling percentage was determined by measuring nitrite concentrations before and after label addition using the Griess method^86^. N_2_ production from denitrification and anammox was calculated from Eqs. (2) and (3), respectively, in Thamdrup et al.^7^ and rates were subsequently calculated from the slope of the linear regression of the N_2_ production as a function of time. If the slope parameter was not significantly different from zero (Student’s *t*-test, *p* < 0.05), then rates were considered as undetectable (i.e. zero).

Areal denitrification and areal anammox rates were calculated by integrating the respective N_2_ production over depth. Only sampling depths with oxygen concentrations below 1.5 µmol L^−1^ were considered. The lowest depth of the integration was either the lower edge of the OMZ or the seafloor. In most cases N_2_ production rates dropped to zero before that depth.

### Statistical correlation tests and linear regressions

All statistical tests were carried out using the R software version 3.6.1^92^. The shapiro.test function was used to test the variables for normality. Spearman’s rank correlation coefficients were calculated to test for correlations, using the cor.test function. Populations were compared either with Student’s *t*-test (function t.test) or Wilcoxen signed rank test (function wilcox.test) depending on normality of the data. Linear regressions were carried out to determine the correlation of anammox rates with particle abundance (particle volume per liter) for the four size classes 128–256, 256–512, 512–1024, 1024–2048 µm. The linear model (lm) function of *R* was used for individual and multiple linear regressions. Figures were made using the ggplot2^93^ package and Adobe Illustrator.

### Collection of sinking organic material

Sinking organic material was collected non-destructively using a Marine Snow Catcher (OSIL, UK) lowered at 1 m s^−1^. The snow catcher was closed in the oxycline or upper part of the OMZ (from oxygen concentrations of 6 µmol L^−1^ down to 150 m below the oxic–anoxic interface, at water depths between 50 and 230 m, dependent on the station; see Supplementary Table 2). The waters of the upper OMZ were previously shown to have the highest N cycling activity and abundance of sinking organic material within the OMZ^5,64^. After retrieval, the snow catcher was placed in an upright position in a shaded area on deck for 1–2 h to allow the sinking organic material to settle to the bottom compartment of the snow catcher and be collected in the snow catcher tray. Subsequently, the overlying water was gently removed at a flow rate of 600 mL min^−1^ and discarded. After this, the snow catcher tray was transferred to the lab and individual particles and aggregates were picked by eye using a wide bore pipette and taking utmost care. The particle or aggregate was transferred to a small glass Petri dish filled with anoxic sterile filtered seawater from the same station. A scaled image was taken of each particle or aggregate for size determination (see below).

### Size determination and categorization of sinking organic material

In order to determine the volume and type of the sinking organic material collected from the snow catcher, scaled images were taken of each particle or aggregate using a SLR-Camera (Nikon D-90) fitted with an AF-S Nikkor zoom lens (*f* = 16–88 mm). Area was determined by manually identifying the edges of the organic material and integrating the inner pixels using a MATLAB script. The volume of each particle was estimated from the equivalent sphere diameter (ESD), assuming a spherical shape. Based on their visual properties, the sinking organic material was categorized manually, following ecotaxa categories where possible (https://ecotaxa.obs-vlfr.fr; ref. ^94^). The main categories were fluffy detritus (loosely packed material), compact detritus (densely packed material), faecal pellets and other sinking material. In addition to the ecotaxa categories, a category for loosely packed detritus containing faecal pellets was created for this dataset.

### Total organic carbon (TOC) content determination of single particles and aggregates

TOC was determined using a combined elemental-analyser/isotopic-ratio mass spectrometer (EA-IRMS, Delta Plus XP coupled to a Flash Element Analyzer 112). Sixty-nine individual particles or aggregates were picked, transferred into sterile-filtered seawater for photography (as described above) and to remove surrounding material, frozen in a droplet of seawater and transported to the laboratory in Eppendorf tubes. After thawing, Eppendorf tubes were centrifuged at 100 rcf for 10 min, and most of the overlying water was removed. The pellet was resuspended in 10–20 µL of the remaining seawater and transferred into a cylindrical 5 × 9 mm tin capsule (HEKAtech GmbH, Germany). Subsequently, the remaining material was collected by rinsing the tube with 10 µL of MilliQ water twice and transferring it to the tin capsule. The ^12^C concentrations of the materials were usually close to the detection limit of the EA-IRMS. In order to increase the signal-intensity, we amended the sample with 4 µg of ^15^N^13^C-labelled urea (99 atom % ^13^C, 98 atom % ^15^N, Sigma Aldrich). The samples were dried in a 60 °C oven and the tin capsules were folded into pellets. Following the same protocol, a standard series was prepared from caffeine with known ^12^C contents ranging between 0 and 12 µg C. Subsequently, the carbon content of the samples and standard series was measured on the EA–IRMS.

### Export production

Export production was estimated based on both satellite imagery and UVP data. Briefly, for the satellite-based estimate chlorophyll *a*, temperature and PAR data for the region were downloaded from Giovanni, NASA (https://giovanni.gsfc.nasa.gov/giovanni/, last visit: 11.05.2020, ref. ^95^) for the time of the cruise. Primary production was calculated based on chlorophyll *a* concentrations using a temperature and photosynthetically active radiation (PAR)-based transfer function^96^. We derived carbon-based export production using a particle export to primary production ratio (*pe*-ratio) determined via two methods: (1) using the global empirical relationship described in ref. ^77^ (based on temperature and chlorophyll a) and (2) using a *pe*-ratio of 0.4 as described in a previous report from the Peruvian OMZ^97^. The UVP-based estimate of export of organic nitrogen out of the euphotic zone was derived for particle size classes between 128 and 2048 µm and was based on the particle flux (*J*) at the base of the euphotic zone and the upper boundary of the OMZ (similar to refs. ^46,98^):

1 $$J={c}_{{\rm{{f}}}}\int_{{r}_{0}}^{{r}_{1}}n\left(r\right)m\left(r\right)U\left(r\right){\rm{d}}r$$

where *n*(*r*) is the particle concentration, *m*(*r*) the size-specific carbon content of the particles, *U*(*r*) the particle settling velocity, *r* is the sphere-equivalent radius of the particles, *r*_0_ and *r*_1_ are the lower and upper limits for integration, respectively, and *c*_f_ is the conversion factor that relates carbon to nitrogen export: *c*_f_ = 1 for carbon export production and *c*_f_ = 0.14 (based on a previously defined C:N ratio of 7.2 in sinking organic matter^5^) for nitrogen export. The measured carbon content (Supplementary Fig. 14) of individual particles was used to rescale the power-law function described by Alldredge 1998 (ref. ^99^):

2 $$m\left(r\right)=8.9V{\left(r\right)}^{0.52}$$

where *m*(*r*) is the carbon content (µg agg^−1^), *V*(*r*) is the total volume of the particle (mm^3^) which was estimated assuming spherical shapes of the particles: *V*(*r*) = 4/3*πr*^3^. The particle settling velocity was calculated based on Stoke’s law assuming that in average the particles are well represented by a spherical shape:

3 $$U\left(r\right)=\frac{2{r}^{2}g}{9\nu }{\rm{\cdot }}\frac{\triangle \rho }{\rho }$$

where *g* is the acceleration by gravity (9.81 m s^−2^), *ν* the kinematic viscosity of sea-water and *ρ* the density of sea-water. *ν* and *ρ* were estimated based on the CTD profiles at the specific depths of the particles. The excess density of the particles was calculated as

4 $$\triangle \rho =\left(1-\theta \right){\rho }_{{\rm{{s}}}}+\rho \left(\theta -1\right)$$

where *ρ*_s_ is the solid-hydrated density (1150 kg m^−3^ for diatom aggregates^100^). The fractal characteristic of marine aggregates leads to a size-dependent porosity^100^ (*r* in mm):

5 $$\theta \left(r\right)=-2.6 \cdot {10}^{-3}{r}^{-1.6}+1$$

### Nitrogen release and encounter rates

In order to investigate the relationship between particle abundance and anammox rates, the UVP data was used to estimate the release of N from particles and the encounter rates of anammox bacteria with the released N. During the descent through the oxic and anoxic water column, ammonium is assumed to be released by remineralization of the organics bound in the particles. We assume that the particle turnover follows a first-order kinetic: d*m*/d*t* = *−R*_rem_*m*, where *R*_rem_ is the carbon-specific degradation rate (day^−1^) and *t* the time variable. Assuming a constant settling velocity of the particle through the water-column, *R*_rem_ can be transformed into a length-scale: *L*_rem_ = *U*/*R*_rem_, the so-called remineralization length-scale. When production, remineralization as well as fragmentation are in balance, the flux attenuation follows an exponential decay:

6 $$J(z)={J}_{0}\cdot {\rm{{{e}}}}^{\left(-\frac{z-{z}_{0}}{{L}_{{\rm{{{rem}}}}}}\right)}$$

where *z* is the depth and *z*_0_ is a reference depth. This equation illustrates the important meaning of *L*_rem_. Substituting *z* = *L*_rem_ immediately yields *J*(*L*_rem_)/*J*_0_ = 0.37, implying that a particle decayed to 37% of its initial value after travelling a distance of *L*_rem_. De Soto et al. (2018) ref. ^101^ derived an extended model for the flux attenuation by introducing the parameter *z*_*β*_ which has the units of depth (m) and can be interpreted as a depth where the degradation of particles decays:

7 $$J(z)={J}_{0}\cdot {\rm{{{e}}}}^{\left[-\frac{{z}_{\beta }}{{L}_{{\rm{{{rem}}}}}}{\rm{\cdot }}\left({\rm{{{e}}}}^{-\frac{z}{{z}_{\beta }}}-\left({{\rm{{e}}}}^{-\frac{{z}_{0}}{{z}_{\beta }}}\right)\right)\right]}$$

We estimated the remineralization length scale by fitting Eqs. (6) and (7) to the carbon flux profiles (Supplementary Fig. 13). Values for *L*_rem_ in the core of the OMZ were found to range between 139 and 305 m with an average of 187 m (Supplementary Table 7) based on Eq. (6) and in between 57 and 195 m with an average of 107 m based on Eq. (7) (Supplementary Table 8). Applying Eq. (3) for the settling velocity, the carbon-specific degradation rates were estimated to range between 0.13 and 0.28 day^−1^ with an average of 0.18 day^−1^ for Eq. (6) and between 0.1 and 0.54 day^−1^ with an average of 0.32 day^−1^ for Eq. (7). Overall, *L*_rem_ as well as *R*_rem_ were in the same range and the models predicted similar trends. Henceforth, we will focus on the estimates based on Eq. (6) as those represent a more conservative range. Based on *L*_rem_, the N released by particles while settling through the water column was estimated as

8 $${{\rm{{NR}}}}={c}_{{\rm{{f}}}}\int_{{r}_{0}}^{{r}_{1}}n\left(r\right)m\left(r\right){R}_{{{\rm{{rem}}}}}\left(r\right){\rm{d}}r$$

Similar to the remineralization length scale, the diffusion length scale indicates the travelled distance of a particle before solutes that are produced in the interior of particles, are released to the water column: *L*_diff_ = *U*/*R*_diff_, where *R*_diff_ is the diffusion timescale estimated as *R*_diff_ = *D*/(*r* + *δ*)^2^ with *D* the diffusion coefficient.

Encounters between the free-living anammox bacteria and the nitrogen that is released into the diffusive boundary layer of the particle were calculated. Therefore, we integrated the diffusive boundary layer area perpendicular to the settling vector over the distance travelled at a given time. Assuming that anammox bacteria are passive tracers in the water column and that the bacteria only need to be near the boundary layer to take up the released N, the encounter rate can be calculated as

9 $${{\rm{{ER}}}}={c}_{{\rm{{S}}}} \int_{{r}_{0}}^{{r}_{1}}n\left(r\right)\pi \left(2r\delta +{\delta }^{2}\right)U\left(r\right){{\rm{d}}r}$$

where *c*_S_ is the concentration of anammox bacteria (4.3 × 10^4^ cells mL^−1^, ref. ^41^) in a given water volume. *δ* = *r*/Sh is the thickness of the diffusive boundary layer which is estimated based on the Sherwood number Sh, Sh = 1 + 0.62·Re^0.41^Sc^0.33^, where Re = *Ur*/*ν* is the Reynolds number and Sc = *v*/*D* the Schmidt number following (refs. ^70,102^). All integrations were performed numerically in Matlab (2018b Mathworks).

## Supplementary information

Supplementary Information

Supplementary Video

Supplementary Tables

Description of Additional Supplementary Files

## Data Availability

Nutrient data from M136 are deposited on Pangaea (10.1594/PANGAEA.904404) in supplement to ref. ^85^. Nutrient data from M138 are deposited on Pangaea (10.1594/PANGAEA.914948). UVP data are available upon request to R. Kiko and will be made available in a data collection to be found at 10.1594/PANGAEA.924375. The UVP data are also freely available via the particle module of ecotaxa at https://ecotaxa.obs-vlfr.fr/part/ (particle projects 25, 95, 96, 97 and 98; please note that usage of ecotaxa requires user registration). The satellite imagery data used in this study was downloaded through the Giovanni online data system, developed and maintained by the NASA GES DISC. All other data are provided within the text or the supplementary material.

## References

[1] Lam P, Kuypers MMM (2011). Microbial nitrogen cycling processes in oxygen minimum zones. Annu. Rev. Mar. Sci..

[2] Codispoti LA (2001). The oceanic fixed nitrogen and nitrous oxide budgets: moving targets as we enter the anthropocene?. Sci. Mar..

[3] Gruber N, Sarmiento JL (1997). Global patterns of marine nitrogen fixation and denitrification. Glob. Biogeochem. Cycles.

[4] Gruber, N. In *The Ocean Carbon Cycle and Climate* (eds Follows, M. & Oguz, T.) 97–148 (Springer Netherlands, 2004).

[5] Kalvelage T (2013). Nitrogen cycling driven by organic matter export in the South Pacific oxygen minimum zone. Nat. Geosci..

[6] Peters BD (2016). Vertical modeling of the nitrogen cycle in the eastern tropical South Pacific oxygen deficient zone using high-resolution concentration and isotope measurements. Glob. Biogeochem. Cycles.

[7] Thamdrup B (2006). Anaerobic ammonium oxidation in the oxygen-deficient waters off northern Chile. Limnol. Oceanogr..

[8] Dalsgaard T, Thamdrup B, Farías L, Revsbech NP (2012). Anammox and denitrification in the oxygen minimum zone of the eastern South Pacific. Limnol. Oceanogr..

[9] Babbin AR, Keil RG, Devol AH, Ward BB (2014). Organic matter stoichiometry, flux, and oxygen control nitrogen loss in the ocean. Science.

[10] Ward BB (2009). Denitrification as the dominant nitrogen loss process in the Arabian Sea. Nature.

[11] Schmidtko S, Stramma L, Visbeck M (2017). Decline in global oceanic oxygen content during the past five decades. Nature.

[12] Stramma L, Johnson GC, Sprintall J, Mohrholz V (2008). Expanding oxygen-minimum zones in the tropical oceans. Science.

[13] Oschlies A, Brandt P, Stramma L, Schmidtko S (2018). Drivers and mechanisms of ocean deoxygenation. Nat. Geosci..

[14] Breitburg, D. et al. Declining oxygen in the global ocean and coastal waters. *Science* **359**, 6371 (2018). 10.1126/science.aam7240 29301986

[15] Cocco V (2013). Oxygen and indicators of stress for marine life in multi-model global warming projections. Biogeosciences.

[16] Stramma L (2012). Expansion of oxygen minimum zones may reduce available habitat for tropical pelagic fishes. Nat. Clim. Chang..

[17] Frölicher TL, Rodgers KB, Stock CA, Cheung WWL (2016). Sources of uncertainties in 21st century projections of potential ocean ecosystem stressors. Glob. Biogeochem. Cycles.

[18] Bopp L (2013). Multiple stressors of ocean ecosystems in the 21st century: projections with CMIP5 models. Biogeosciences.

[19] Capone DG, Hutchins DA (2013). Microbial biogeochemistry of coastal upwelling regimes in a changing ocean. Nat. Geosci..

[20] Bianchi D, Weber TS, Kiko R, Deutsch C (2018). Global niche of marine anaerobic metabolisms expanded by particle microenvironments. Nat. Geosci..

[21] Niemeyer D, Kriest I, Oschlies A (2019). The effect of marine aggregate parameterisations on nutrients and oxygen minimum zones in a global biogeochemical model. Biogeosciences.

[22] Woebken D, Fuchs BM, Kuypers MMM, Amann R (2007). Potential interactions of particle-associated anammox bacteria with bacterial and archaeal partners in the Namibian upwelling system. Appl. Environ. Microbiol..

[23] Ganesh S (2015). Size-fraction partitioning of community gene transcription and nitrogen metabolism in a marine oxygen minimum zone. ISME J..

[24] Suter EA, Pachiadaki M, Taylor GT, Astor Y, Edgcomb VP (2018). Free-living chemoautotrophic and particle-attached heterotrophic prokaryotes dominate microbial assemblages along a pelagic redox gradient. Environ. Microbiol..

[25] Fuchsman CA, Devol AH, Saunders JK, McKay C, Rocap G (2017). Niche partitioning of the N cycling microbial community of an offshore oxygen deficient zone. Front. Microbiol..

[26] Ganesh S, Parris DJ, DeLong EF, Stewart FJ (2014). Metagenomic analysis of size-fractionated picoplankton in a marine oxygen minimum zone. ISME J..

[27] Riley, J. S. et al. The relative contribution of fast and slow sinking particles to ocean carbon export. *Glob. Biogeochem. Cycles* **26**, 1 (2012).

[28] Walker BD, Beaupré SR, Guilderson TP, McCarthy MD, Druffel ERM (2016). Pacific carbon cycling constrained by organic matter size, age and composition relationships. Nat. Geosci..

[29] Benner R, Amon RMW (2015). The size-reactivity continuum of major bioelements in the ocean. Annu. Rev. Mar. Sci..

[30] Cavan EL, Trimmer M, Shelley F, Sanders R (2017). Remineralization of particulate organic carbon in an ocean oxygen minimum zone. Nat. Commun..

[31] Iversen MH, Ploug H (2010). Ballast minerals and the sinking carbon flux in the ocean: carbon-specific respiration rates and sinking velocity of marine snow aggregates. Biogeosciences.

[32] Weber T, Cram JA, Leung SW, DeVries T, Deutsch C (2016). Deep ocean nutrients imply large latitudinal variation in particle transfer efficiency. Proc. Natl Acad. Sci. USA.

[33] Maerz J, Six KD, Stemmler I, Ahmerkamp S, Ilyina T (2020). Microstructure and composition of marine aggregates as co-determinants for vertical particulate organic carbon transfer in the global ocean. Biogeosciences.

[34] Callbeck CM, Lavik G, Stramma L, Kuypers MMM, Bristow LA (2017). Enhanced nitrogen loss by eddy-induced vertical transport in the offshore Peruvian oxygen minimum zone. PLoS ONE.

[35] Pennington JT (2006). Primary production in the eastern tropical Pacific: a review. Prog. Oceanogr..

[36] Rodríguez-Morata C, Díaz HF, Ballesteros-Canovas JA, Rohrer M, Stoffel M (2019). The anomalous 2017 coastal El Niño event in Peru. Clim. Dyn..

[37] Garreaud RD (2018). A plausible atmospheric trigger for the 2017 coastal El Niño. Int. J. Climatol..

[38] Fi, O.-G. Coastal el niño 2017 or simply: the carnival coastal warming event? *MOJES* **2**, 8 (2017).

[39] Echevin, V. et al. Forcings and evolution of the 2017 coastal El Niño off northern Peru and Ecuador. *Front. Mar. Sci*. **5**, 367 (2018).

[40] Callbeck CM (2018). Oxygen minimum zone cryptic sulfur cycling sustained by offshore transport of key sulfur oxidizing bacteria. Nat. Commun..

[41] Hamersley MR (2007). Anaerobic ammonium oxidation in the Peruvian oxygen minimum zone. Limnol. Oceanogr..

[42] Picheral M (2010). The Underwater Vision Profiler 5: an advanced instrument for high spatial resolution studies of particle size spectra and zooplankton. Limnol. Oceanogr. Methods.

[43] Sheldon RW, Prakash A, Sutcliffe WH (1972). The size distribution of particles in the ocean1. Limnol. Oceanogr..

[44] Stemmann L, Boss E (2012). Plankton and particle size and packaging: from determining optical properties to driving the biological pump. Annu. Rev. Mar. Sci..

[45] Kiko R (2017). Biological and physical influences on marine snowfall at the equator. Nat. Geosci..

[46] Guidi L (2008). Relationship between particle size distribution and flux in the mesopelagic zone. Deep Sea Res. Part I.

[47] Baker CA (2017). Slow-sinking particulate organic carbon in the Atlantic Ocean: magnitude, flux, and potential controls. Glob. Biogeochem. Cycles.

[48] Engel, A., Endres, S., Galgani, L. & Schartau, M. Marvelous marine microgels: on the distribution and impact of gel-like particles in the oceanic water-column. *Front. Mar. Sci*. **7**, 405 (2020).

[49] Iversen, M. H. & Lampitt, R. S. Size does not matter after all: no evidence for a size-sinking relationship for marine snow. *Prog. Oceanogr*. 102445 (2020).

[50] Cavan EL, Giering SLC, Wolff GA, Trimmer M, Sanders R (2018). Alternative particle formation pathways in the eastern tropical north pacific’s biological carbon pump. J. Geophys. Res. Biogeosci..

[51] Briggs N, Dall’Olmo G, Claustre H (2020). Major role of particle fragmentation in regulating biological sequestration of CO2 by the oceans. Science.

[52] Kiko, R. et al. Zooplankton-mediated fluxes in the Eastern Tropical North Atlantic. *Front. Mar. Sci*. **7**, 353 (2020).

[53] Bianchi D, Babbin AR, Galbraith ED (2014). Enhancement of anammox by the excretion of diel vertical migrators. Proc. Natl Acad. Sci. USA.

[54] Galán A, Faúndez J, Thamdrup B, Santibáñez JF, Farías L (2014). Temporal dynamics of nitrogen loss in the coastal upwelling ecosystem off central Chile: evidence of autotrophic denitrification through sulfide oxidation. Limnol. Oceanogr..

[55] Lam P (2009). Revising the nitrogen cycle in the Peruvian oxygen minimum zone. Proc. Natl Acad. Sci. USA.

[56] Galán A (2009). Anammox bacteria and the anaerobic oxidation of ammonium in the oxygen minimum zone off northern Chile. Deep Sea Res. Part II.

[57] Ward BB (2008). Organic carbon, and not copper, controls denitrification in oxygen minimum zones of the ocean. Deep Sea Res. Part I.

[58] Fuchsman CA, Staley JT, Oakley BB, Kirkpatrick JB, Murray JW (2012). Free-living and aggregate-associated Planctomycetes in the Black Sea. FEMS Microbiol. Ecol..

[59] Lavik G (2009). Detoxification of sulphidic African shelf waters by blooming chemolithotrophs. Nature.

[60] Callbeck, C. M. et al. *Arcobacter peruensis* sp. nov., a chemolithoheterotroph isolated from sulfide- and organic-rich coastal waters off Peru. *Appl. Environ. Microbiol*. **85**, 24 (2019). 10.1128/AEM.01344-19 PMC6881792 31585991

[61] Hach PF (2020). Rapid microbial diversification of dissolved organic matter in oceanic surface waters leads to carbon sequestration. Sci. Rep..

[62] Martin JH, Knauer GA, Karl DM, Broenkow WW (1987). VERTEX: carbon cycling in the northeast Pacific. Deep Sea Res. Part A.

[63] Van Mooy BA, Keil RG, Devol AH (2002). Impact of suboxia on sinking particulate organic carbon: enhanced carbon flux and preferential degradation of amino acids via denitrification. Geochim. Cosmochim. Acta.

[64] Kalvelage T (2015). Aerobic microbial respiration in oceanic oxygen minimum zones. PLoS ONE.

[65] De Brabandere L (2014). Vertical partitioning of nitrogen-loss processes across the oxic-anoxic interface of an oceanic oxygen minimum zone. Environ. Microbiol..

[66] Garcia-Robledo E (2017). Cryptic oxygen cycling in anoxic marine zones. Proc. Natl Acad. Sci. USA.

[67] Srain BM (2020). Fermentation and anaerobic oxidation of organic carbon in the oxygen minimum zone of the upwelling ecosystem off concepción, in Central Chile. Front. Mar. Sci..

[68] Jackson GA (2012). Seascapes: the world of aquatic organisms as determined by their particulate natures. J. Exp. Biol..

[69] Zetsche, E., Larsson, A. I., Iversen, M. H. & Ploug, H. Flow and diffusion around and within diatom aggregates: effects of aggregate composition and shape. *Limnol. Oceanogr*. **65**, 8 (2020).

[70] Kiørboe T (2001). Formation and fate of marine snow: small-scale processes with large- scale implications. Sci. Mar..

[71] Kiørboe T, Jackson GA (2001). Marine snow, organic solute plumes, and optimal chemosensory behavior of bacteria. Limnol. Oceanogr..

[72] Stocker R, Seymour JR, Samadani A, Hunt DE, Polz MF (2008). Rapid chemotactic response enables marine bacteria to exploit ephemeral microscale nutrient patches. Proc. Natl Acad. Sci. USA.

[73] Stocker R (2012). Marine microbes see a sea of gradients. Science.

[74] Strous M (1999). Missing lithotroph identified as new planctomycete. Nature.

[75] Kuypers MMM (2003). Anaerobic ammonium oxidation by anammox bacteria in the Black Sea. Nature.

[76] van Niftrik L, Jetten MSM (2012). Anaerobic ammonium-oxidizing bacteria: unique microorganisms with exceptional properties. Microbiol. Mol. Biol. Rev..

[77] Zhao, R. et al. Geochemical transition zone powering microbial growth in subsurface sediments. *Proc. Natl. Acad. Sci.* **117**, 51 (2020). 10.1073/pnas.2005917117 PMC7768721 33288718

[78] Woebken D (2008). A microdiversity study of anammox bacteria reveals a novel Candidatus Scalindua phylotype in marine oxygen minimum zones. Environ. Microbiol..

[79] Lambert BS, Fernandez VI, Stocker R (2019). Motility drives bacterial encounter with particles responsible for carbon export throughout the ocean. Limnol. Oceanogr..

[80] Codispoti LA, Packard TT (1980). Denitrification rates in the eastern tropical South Pacific. J. Mar. Res..

[81] Deutsch C, Gruber N, Key RM, Sarmiento JL, Ganachaud A (2001). Denitrification and N_2_ fixation in the Pacific Ocean. Glob. Biogeochem. Cycles.

[82] DeVries T, Deutsch C, Primeau F, Chang B, Devol A (2012). Global rates of water-column denitrification derived from nitrogen gas measurements. Nat. Geosci..

[83] Lombard, F. et al. Globally consistent quantitative observations of planktonic ecosystems. *Front. Mar. Sci*. **6**, 196 (2019).

[84] Giering, S. L. C. et al. Sinking organic particles in the ocean—flux estimates from in situ optical devices. *Front. Mar. Sci*. **6**, 834 (2020).

[85] Lüdke, J. et al. *Influence of Intraseasonal Eastern Boundary Circulation Variability on Hydrography and Biogeochemistry off Peru* (PANGAEA, 2019).

[86] Grasshoff, K., Kremling, K. & Ehrhardt, M. *Methods of Seawater Analysis* (Wiley, 1999).

[87] Loginova AN, Thomsen S, Engel A (2016). Chromophoric and fluorescent dissolved organic matter in and above the oxygen minimum zone off Peru. J. Geophys. Res. Oceans.

[88] Hydes, D. et al. in *The GO-SHIP Repeat Hydrography Manual: A Collection of Expert Reports and Guidelines*. *IOCCP Report No. 14, ICPO Publication Series No. 134, Version 1, 2010* (eds Hood, E. M., C. L. Sabine & B. M. Sloyan) (UNESCO/IOC, 2010).

[89] Holmes RM, Aminot A, Kérouel R, Hooker BA, Peterson BJ (1999). A simple and precise method for measuring ammonium in marine and freshwater ecosystems. Can. J. Fish. Aquat. Sci..

[90] Dalsgaard T, Canfield DE, Petersen J, Thamdrup B, Acuña-González J (2003). N2 production by the anammox reaction in the anoxic water column of Golfo Dulce, Costa Rica. Nature.

[91] De Brabandere L, Thamdrup B, Revsbech NP, Foadi R (2012). A critical assessment of the occurrence and extend of oxygen contamination during anaerobic incubations utilizing commercially available vials. J. Microbiol. Methods.

[92] Team, R. C. & DC, R. *A Language and Environment For Statistical Computing* (R Foundation for Statistical Computing, 2019).

[93] Wickham, H. *ggplot2—Elegant Graphics for Data Analysis* (Springer International Publishing, 2016).

[94] Picheral, M., Colin, S. & Irisson, J. O. *EcoTaxa, A Tool for the Taxonomic Classification of Images* http://ecotaxa.obs-vlfr.fr (2017).

[95] Acker JG, Leptoukh G (2007). Online analysis enhances use of NASA earth science data. EOS Trans. AGU.

[96] Behrenfeld MJ, Falkowski PG (1997). Photosynthetic rates derived from satellite-based chlorophyll concentration. Limnol. Oceanogr..

[97] Dunne, J. P., Armstrong, R. A., Gnanadesikan, A. & Sarmiento, J. L. Empirical and mechanistic models for the particle export ratio. *Glob. Biogeochem. Cycles* **19**, 4 (2005).

[98] Iversen MH, Nowald N, Ploug H, Jackson GA, Fischer G (2010). High resolution profiles of vertical particulate organic matter export off Cape Blanc, Mauritania: degradation processes and ballasting effects. Deep Sea Res. Part I.

[99] Alldredge A (1998). The carbon, nitrogen and mass content of marine snow as a function of aggregate size. Deep Sea Res. Part I.

[100] Alldredge AL, Gotschalk C (1988). In situ settling behavior of marine snow. Limnol. Oceanogr..

[101] De Soto F, Ceballos-Romero E, Villa-Alfageme M (2018). A microscopic simulation of particle flux in ocean waters: application to radioactive pair disequilibrium. Geochim. Cosmochim. Acta.

[102] Ploug H, Hietanen S, Kuparinen J (2002). Diffusion and advection within and around sinking, porous diatom aggregates. Limnol. Oceanogr..

