# Supplementary material for: Small sinking particles control anammox rates in the Peruvian oxygen minimum zone
Source: Nat Commun. 2021 May 28;12:3235. doi: 10.1038/s41467-021-23340-4 (PMC8163745; doi:10.1038/s41467-021-23340-4)
Supplement: Supplementary file 1 — Supplementary Information [file 41467_2021_23340_MOESM1_ESM.pdf]

Supplements: Small sinking particles control anammox rates in the Peruvian oxygen minimum zone

Clarissa Karthäuser*[1], Soeren Ahmerkamp*,[1,2], Hannah K Marchant**[1,2], Laura A Bristow[1,3], Helena Hauss[4], Morten H Iversen[2,5], Rainer Kiko[4,6], Gaute Lavik[1], Joeran Maerz[7], Marcel MM Kuypers[1]**

1 Max Planck Institute for Marine Microbiology, Bremen, Germany

2 MARUM - Center for Marine Environmental Sciences, Bremen, Germany

3 Current address: Department of Biology, University of Southern Denmark, Odense, Denmark

4 GEOMAR Helmholtz Center for Ocean Research Kiel, Kiel, Germany

5 Alfred Wegener Institute Helmholtz Center for Polar and Marine Research, Bremerhaven, Germany

6 Current address: Laboratoire d'Océanographie de Villefranche-sur-Mer, France

7 Max Planck Institute for Meteorology, Hamburg, Germany

*** Equal contribution**

**** Corresponding authors**

Supplementary Table 1 and 2 are provided as spreadsheets (Supplementary_Data.xlsx).

Supplementary Table 3: Categorization of particles types within different size classes of material collected with the Marine Snow Catcher. The aggregates were categorized manually according to their visual properties using the Ecotaxa categories as a guideline (https://ecotaxa.obs-vlfr.fr; ref.[1]).

	128 - 256 µm	256 - 512 µm	512 - 1024 µm	1024 - 2048 µm
loose detritus	28 %	46 %	56 %	27 %
compact detritus	44 %	33 %	13 %	3 %
detritus containing fecal pellets	0 %	1 %	17 %	59 %
fecal pellets	8 %	6 %	3 %	1 %
other	19 %	15 %	11 %	11 %
total number	36	448	351	75

Supplementary Table 4: Correlations between anammox rates in the size fractionated water samples (Figure 3).

Compared fractions	Spearman ρ (rho)	Spearman p*	Paired t-test	T-test p	df
Bulk and 1.6 µm	0.65	0.00032	0.20	0.84	25
Bulk and 10 µm	0.67	0.00016	-0.90	0.38	25
10 µm and 1.6 µm	0.47	0.014	-0.78	0.44	26

*A Spearman p-value < 0.05 indicates that there was a significant correlation at a 95 % confidence interval

Supplementary Table 5: Statistics for correlations between anammox rates and *in situ* particle abundances during April 2017 (Figure 4). For each size class, a Spearman's correlation test was carried out using all anammox rates (if $p > 0.05$, the rate was assumed as zero) from anoxic water depths and corresponding particle abundances measured using a UVP5 (which was attached to the same CTD that was used to sample water for the rate determinations). A linear regression was carried out between particle abundances or biovolumes in each size class and anammox rates. The sample size was $n = 24$. In all cases, one anammox rate and associated particle abundances from the euphotic zone (24 m depth, station 341) was excluded as it was > 6 SD from the mean. All correlation tests were carried out using the linear model (lm) function of R.

	**Size Class**	**Spearman's p-value**	**Spearman's ρ (rho)**	**R^2**	**Slope**	**Intercept**
Biovolume	128 - 256 µm	$1.1 \cdot 10^{-9}$	0.91	0.81	390	-15
	256 - 512 µm	$5.9 \cdot 10^{-8}$	0.86	0.55	140	-0.15
	512 - 1024 µm	0.0046	0.56	0.23	39	15
	1024 - 2048 µm	0.048	0.41	0.28	58	12
Number	128 - 256 µm	$2.0 \cdot 10^{-9}$	0.90	0.82	0.86	-15.9
	256 - 512 µm	$1.2 \cdot 10^{-8}$	0.88	0.60	3.7	-1.87
	512 - 1024 µm	0.00076	0.64	0.30	8.5	11.1
	1024 - 2048 µm	0.011	0.51	0.32	72.8	11.4

Supplementary table 6: Multiple linear regression for the correlation of anammox rates with total particle volumes within the four size classes.

Correlations between total particle abundance and anammox rates for each individual size class (models S = 128-256 µm, M = 256-512 µm, L = 512-1024 µm, XL = 1024-2048 µm), showing that the correlation was strongest for the smallest size class (128 - 256 µm). To test whether the inclusion of multiple size classes would improve the correlation between particle volume and anammox rates, different combinations of size classes were used in a multiple linear regression approach (models 1 and 2). In model 1 (all four size classes), size class M and XL were not significant to predict anammox. Especially in the case of size class M this was due to the collinearity with size class S (R^2 = 0.72). Therefore size class M and XL were removed to calculate model 2. Compared with estimating anammox from only size class S, the potential of the model to predict anammox was slightly improved by including size class L (AIC changed from 190 to 188, BIC stayed the same, as BIC penalizes model complexity more than AIC). Therefore we decided not to include another size class to estimate anammox rates, as the effect is sufficiently explained by size class S and the contributing factor of size class L is small compared to S. Also, size class S was best at explaining the variation in the available data, as can be seen by its high F statistic and p-value.

	**Model**	**a**	**b**	**c**	**d**	**int**	**F**	**P value**	**sigma**	**df**	**R^2 (adj.)**	**AIC**	**BIC**
1	AMX = a·S + b·M + c·L + d·XL + int	471	-62	21	1.4	-19	30	$6 \cdot 10^{-8}$	10.8	19	0.83	189	196
	Error	89	42	11	13	5							
	T value	5	-2	2	0.1	-4							
	Pr(>|t|)	$4 \cdot 10^{-5}$	0.15	0.07	0.9	0.001							
2	AMX = a·S + c·L + int	366		14		-18	55	$4.7 \cdot 10^{-9}$	11.1	21	0.82	188	193
	Error	41		8		5.2							
	T value	9		1.8		-3.4							
	Pr(>|t|)	$1 \cdot 10^{-8}$		0.09		0.003							
S	AMX = a·S + int	393				-15	96	$1.7 \cdot 10^{-9}$	11.6	22	0.81	190	193
	Error	40				5							
	T value	10				-2.9							
	Pr(>|t|)	$2 \cdot 10^{-9}$				0.009							
M	AMX = b·M + int		143			-0.2	27	$3 \cdot 10^{-5}$	18.0	22	0.55	211	214
	Error		27			7							
	T value		5			-0.02							
	Pr(>|t|)		$3 \cdot 10^{-5}$			0.98							
L	AMX = c·L + int			39		15.5	6.4	0.02	23.7	22	0.19	224	228
	Error			16		8							
	T value			2.5		2							
	Pr(>|t|)			0.02		0.06							
XL	AMX = d·XL + int				58	11.9	8.5	0.008	22.9	22	0.28	222	226
	Error				20	8.1							
	T value				3	1.5							
	Pr(>|t|)				0.008	0.15							

Supplementary Table 7: Remineralization of particles determined from the carbon flux profiles shown in Supplementary Figure 2 using a simple exponential decay (equation 6, see also ref. [2]). Remineralization length scales are calculated based on the carbon flux attenuation profiles (Supplementary Figure 15). The remineralization length scale (L_{rem}) indicates at which depth the particle decays to 37% of its initial value and can be used to estimate the degradation rate (R_{rem}). Based on the degradation rate and by considering the carbon content in particles, the remineralization rate (dm/dt) is estimated.

	**Remineralization Euphotic base**			**Remineralization OMZ ***			**Remineralization OMZ - 50 ****		
	L_{rem} (m)	R_{rem} (d^{-1})	dm/dt (µmol C L^{-1} d^{-1})	L_{rem} (m)	R_{rem} (d^{-1})	dm/dt (µmol C L^{-1} d^{-1})	L_{rem} (m)	R_{rem} (d^{-1})	dm/dt (µmol C L^{-1} d^{-1})
Onshore 128 - 512 µm	83	0.28	1.9	50	0.48	1.6	157	0.15	0.5
Onshore 512 - 2048 µm	81	0.5	1.3	51	0.81	0.4	146	0.28	0.14
Offshore 128 - 512 µm	12	2.04	4.7	323	0.07	0.1	139	0.17	0.26
Offshore 512 - 2048 µm	35	1.16	1.3	67	0.61	0.24	305	0.13	0.05

* The depth where oxygen dropped below < 1.5 µM

** 50 m below the depth where oxygen dropped below < 1.5 µM

Supplementary Table 8: Remineralization of particles determined from the carbon flux profiles shown in Supplementary Figure 2 based on the de Soto et al. 2018[3] approach (equation 7). Z_β represents the characteristic depths where the degradation declines. The remineralization length scale (L_{rem}), degradation rate (R_{rem}) and remineralization rate (dm/dt) are shown. The remineralization rate is calculated based on the carbon content in particles, their abundance and the degradation rate.

	**Remineralization Euphotic base**				**Remineralization OMZ***			
	L_{rem} (m)	R_{rem} (d^{-1})	dm/dt (µmol C L^{-1} d^{-1})	Z_β (m)	L_{rem} (m)	R_{rem} (d^{-1})	dm/dt (µmol C L^{-1} d^{-1})	Z_β (m)
Onshore Stations 128 µm - 512 µm	55	0.36	2.4	122	195	0.1	0.3	500
Onshore Stations 512 µm - 2048 µm	47	0.71	1.8	143	112	0.3	0.2	164
Offshore Stations 128 µm - 512 µm	59	0.33	0.8	455	57	0.34	0.5	204
Offshore Stations 512 µm - 2048 µm	59	0.58	0.7	823	62	0.54	0.2	220

Supplementary Table 9: Correlations between organic nitrogen export out of the euphotic zone and integrated anammox rates - see Supplementary Figure 6. A Spearman's correlation test was carried out for nitrogen export estimated from UVP measurements and integrated anammox rates (if p > 0.05, the rate was assumed as zero) from anoxic water depths (where O_2 < 1.5 µM). A linear regression was carried out between particle abundances or biovolumes in each size class and anammox rates. The sample size was n= 24.

		**Spearman's p-value**	**Spearman's ρ (rho)**	**slope**	**intercept**
a	Anammox ~ UVP export	0.04	0.57	0.14	-0.44
b	Anammox ~ Satellite global	0.03	0.60	0.13	1.33
c	Anammox ~ Satellite regional	0.04	0.58	0.11	1.03
d	N loss ~ UVP export	0.01	0.71	0.14	-0.10
e	N loss ~ Satellite global	0.05	0.56	0.11	1.81
f	N loss ~ Satellite regional	0.06	0.53	0.09	1.54

Supplementary Table 10: Statistics for anammox - particle encounter correlations (Supplementary Figure 17). Spearman's correlation test on all anammox rates (if p > 0.05, the rate was assumed as zero) from anoxic samples from M136 (the UVP was attached directly to CTD in all cases). The sample size was n= 24, excluding an outlier that was from the euphotic zone (24m depth, station 341). A linear regression was carried out between anammox bacteria - particle encounters in each size class and anammox rates.

**Size Class**	**Spearman's p-value**	**Spearman's ρ (rho)**	**R^2**	**Slope**	**Intercept**
128 - 256 µm	$1.2 \cdot 10^{-8}$	0.88	0.82	$2.3 \cdot 10^{-5}$	-14.5
256 - 512 µm	$6.4 \cdot 10^{-8}$	0.86	0.58	$1.7 \cdot 10^{-5}$	-2.0
512 - 1024 µm	0.0056	0.55	0.21	$0.73 \cdot 10^{-5}$	15.9
1024 - 2048 µm	0.0089	0.54	0.32	$0.20 \cdot 10^{-5}$	8.3

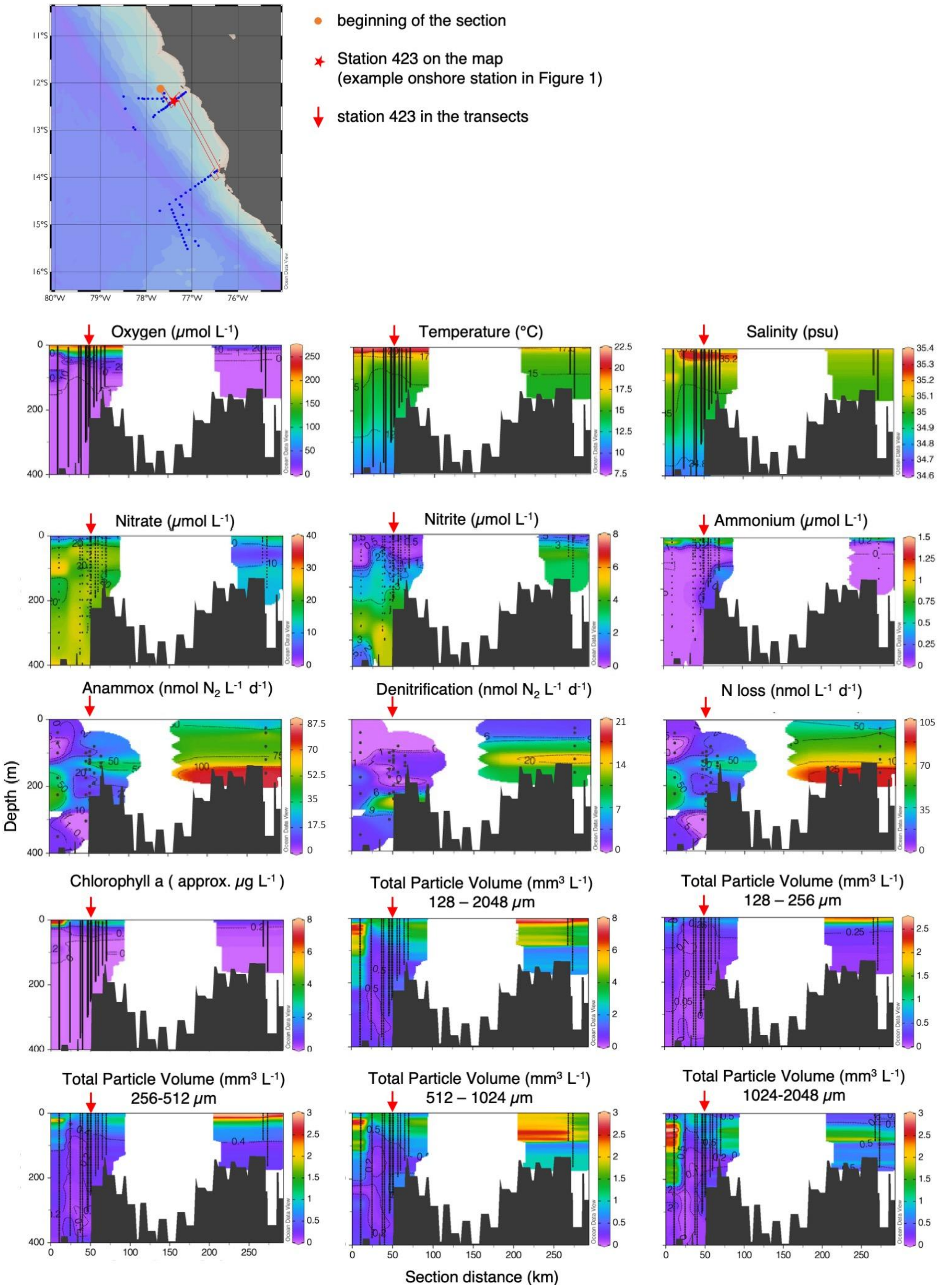

Supplementary Figure 1a: Physico-chemical data from onshore stations during April 2017 (cruise M136).

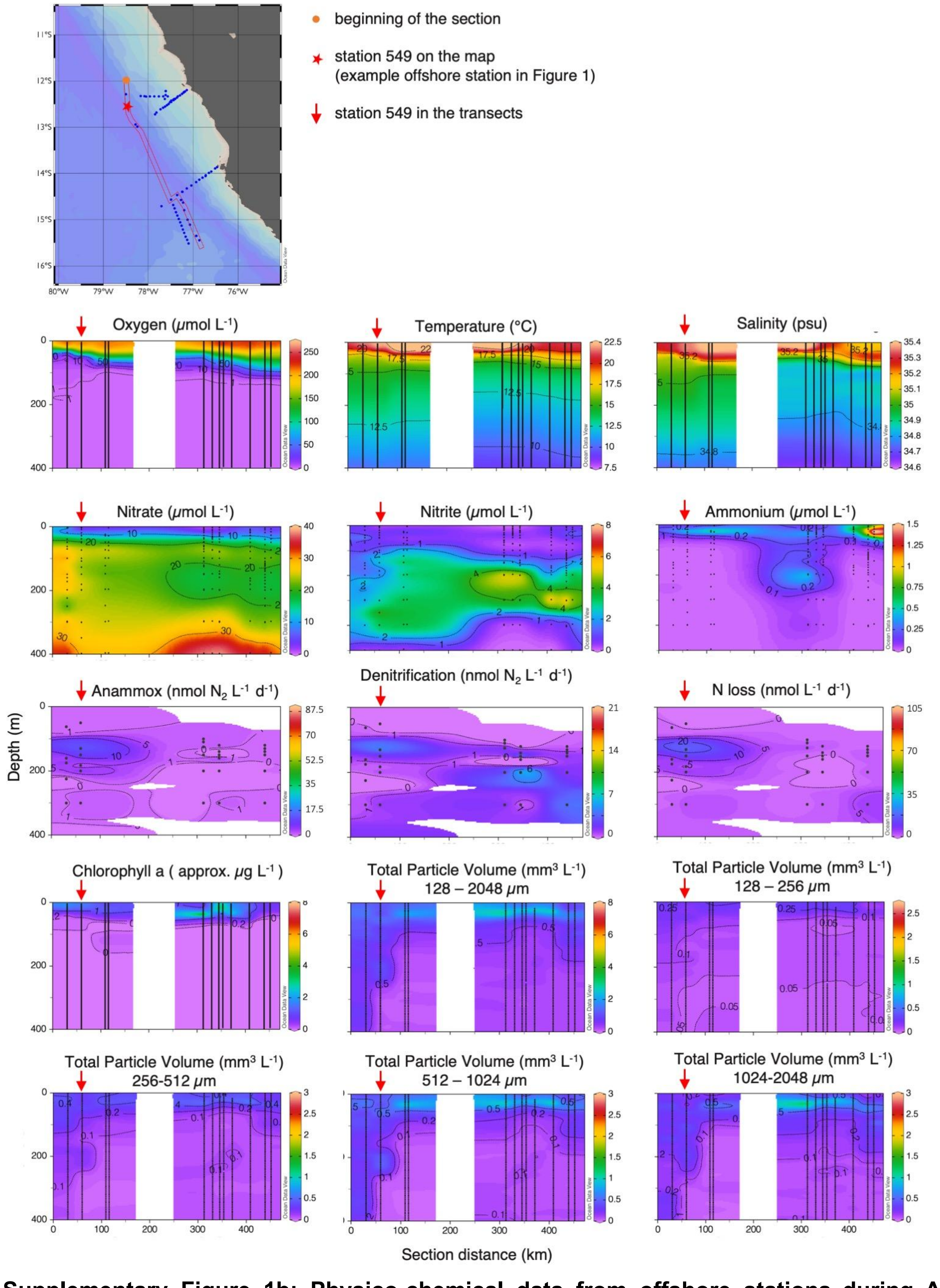

Supplementary Figure 1b: Physico-chemical data from offshore stations during April 2017 (cruise M136).

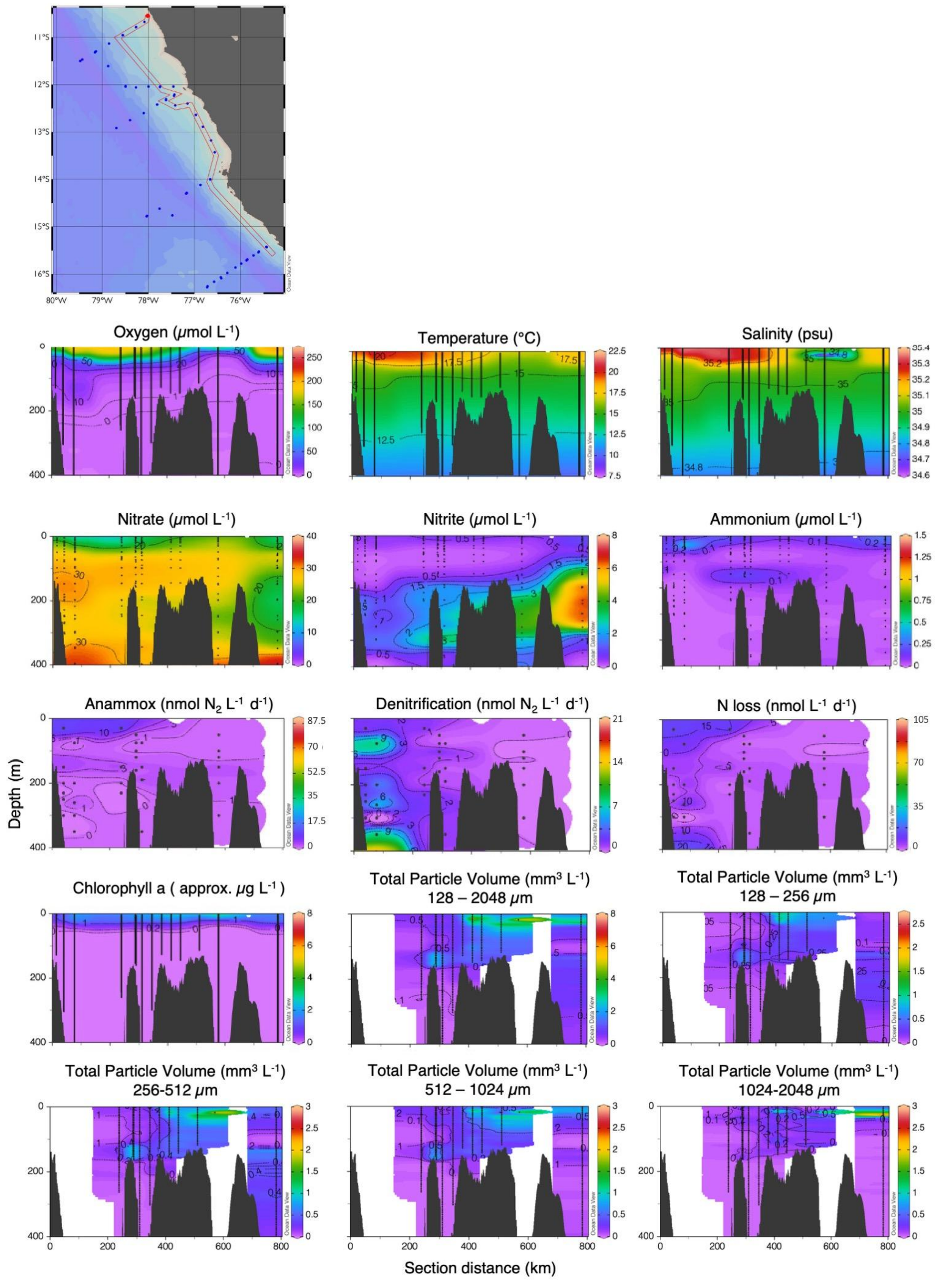

Supplementary Figure 1c: Physico-chemical data from onshore stations during June 2017 (cruise M138).

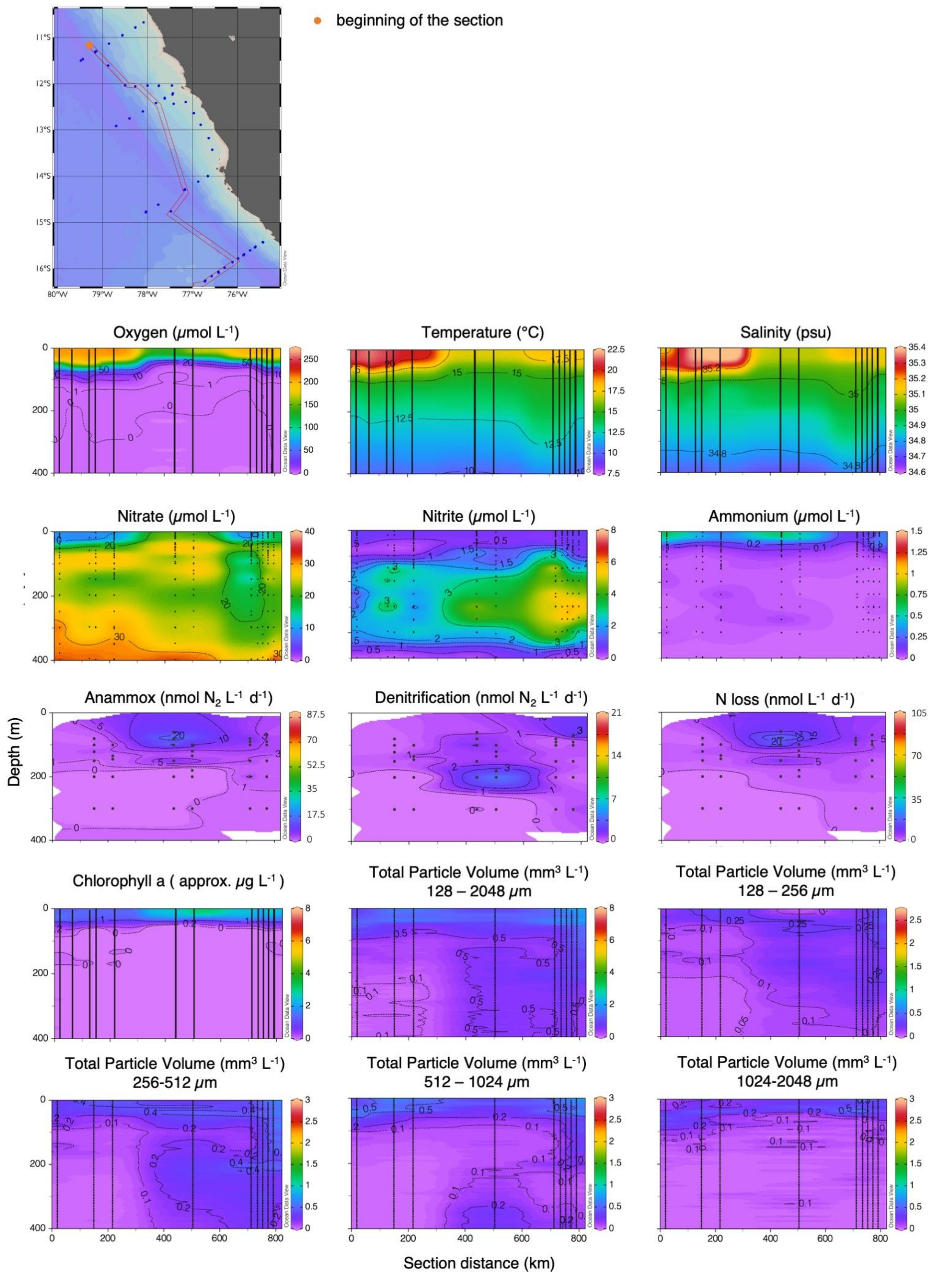

Supplementary Figure 1d: Physico-chemical data from offshore stations during June 2017 (cruise M138).

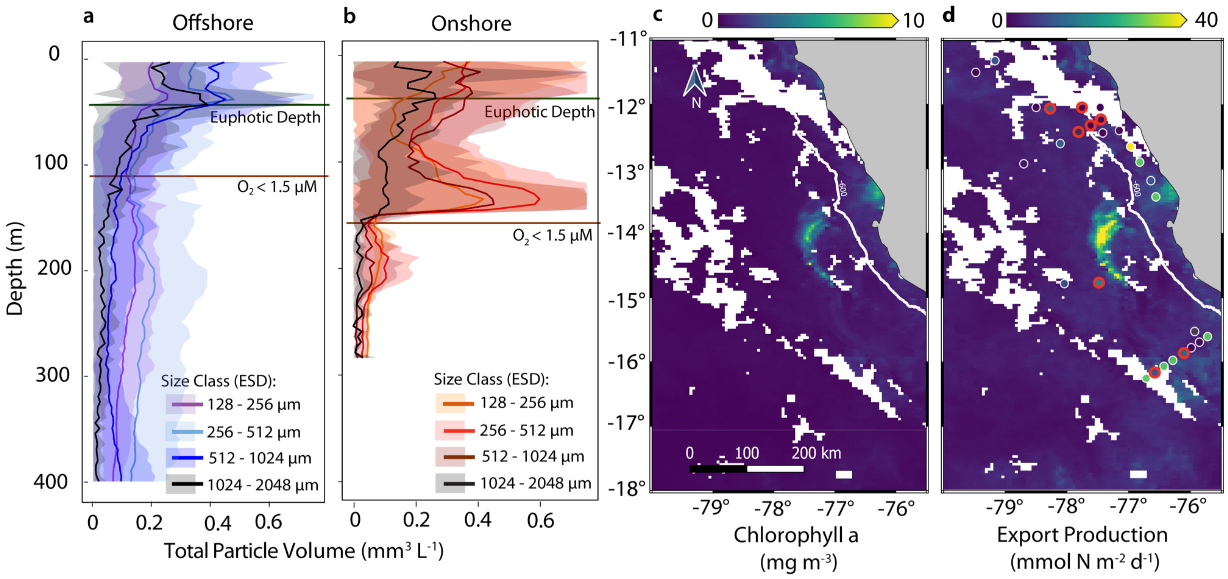

Supplementary Figure 2: Total particle volume within the size classes and export production in the Peruvian upwelling system during June 2017 (M138). Average particle abundance profiles in **a** offshore and **b** onshore stations determined by UVP. Particles were sorted into four size classes with equivalent spherical diameters (ESD) of 128 – 256 µm, 256 – 512 µm, 512 -1024 µm, and 1 – 2 mm. The shaded envelopes correspond to standard deviation in abundances from 20 offshore and 15 onshore stations. They were cropped at 2 mm^3 L^{-1}. The base of the euphotic zone (PAR < 1%, based on satellite data) and the oxic-anoxic interface (where O_2 dropped to < 1 µM) are indicated. **c** Average chlorophyll *a* concentrations from satellite data. **d** Export production estimated from satellite products (pseudocolor map) and from particle abundances at the base of the euphotic zone (circles, see text). The white line is the 600 m isobath on which basis the onshore and offshore stations were separated. Stations where anammox incubations were performed are circled in red.

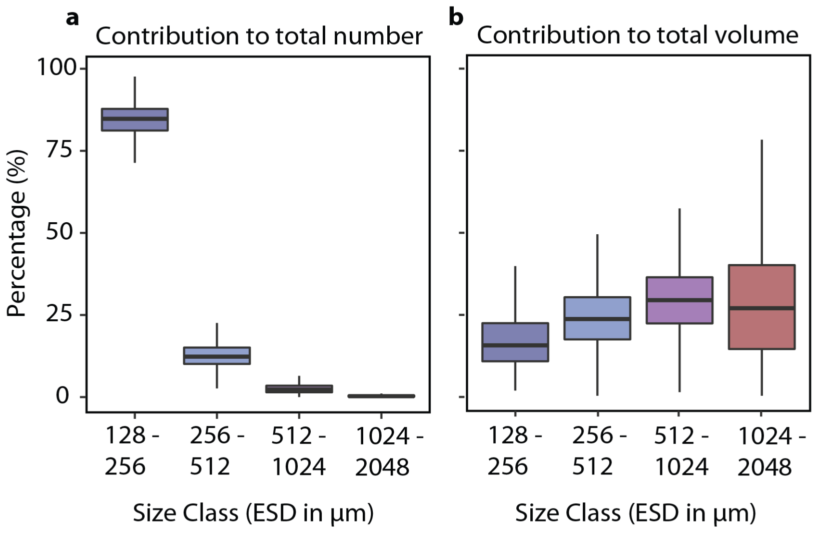

Supplementary Figure 3: Distribution of particle number and volume within the chosen bins for the UVP size classes. Boxplots depict the 25–75 % quantile range, with the center line depicting the median (50 % quantile); whiskers encompass data points within 1.5 times the interquartile range.

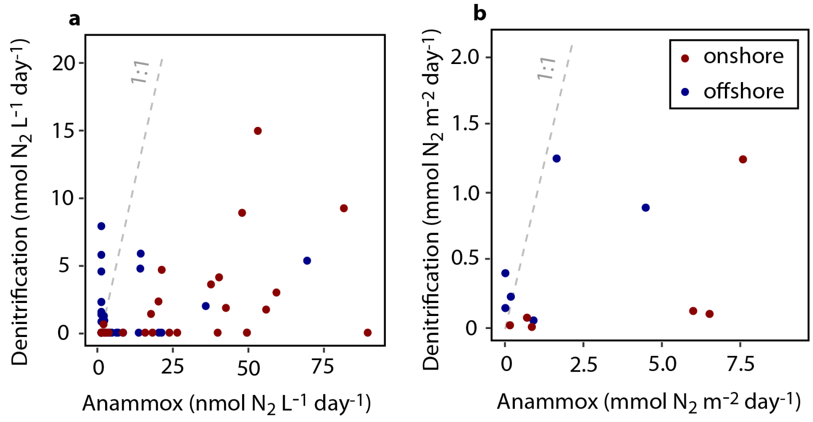

Supplementary Figure 4: Ratio between anammox and denitrification rates determined on both M136 and M138. **a** Volumetric rates, **b** integrated rates at each station. The dashed line represents a 1:1 relationship, therefore any value below this line is representative of a depth or station where anammox rates were higher than denitrification rates.

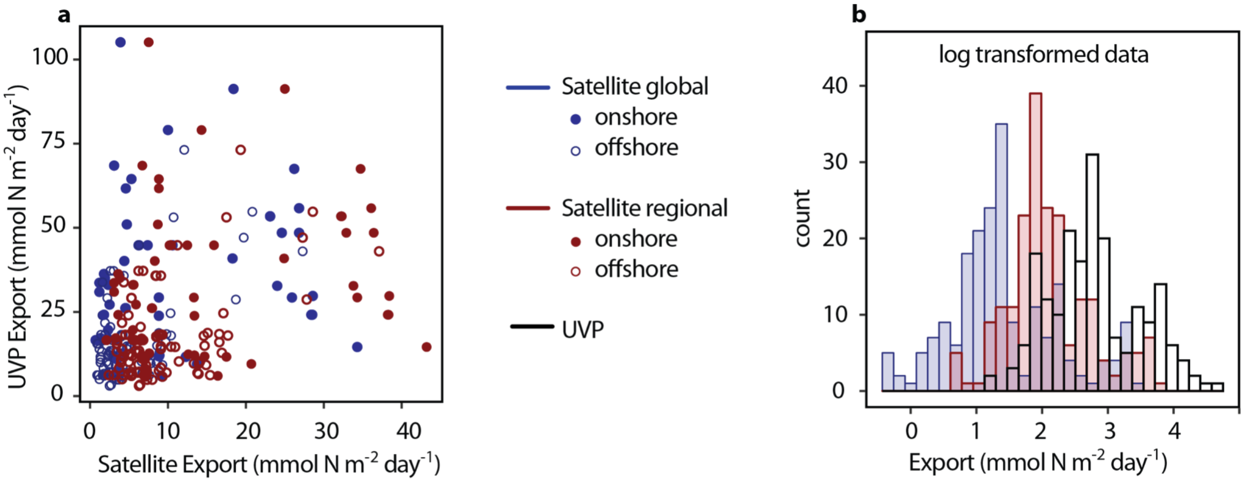

Supplementary Figure 5: Comparison of organic nitrogen export from the photic zone estimated from satellite imaging and UVP profiles. Organic nitrogen export was estimated from the UVP based on particle concentration at the base of the euphotic zone, measured size specific carbon content of the particles and calculated particle settling velocity (black). Organic nitrogen export estimates from satellite data were calculated using two different methods, firstly based on calculating a primary production export-ratio from temperature and chlorophyll concentrations[4] (red) and secondly based on a regional regional estimate of export to primary production ratio (blue). Correlations for all individual data points are given in **a** and distribution of values is shown in **b**, histogram showing the distribution of organic N export based on the three different methods, organic nitrogen export estimates were binned into classes of 0.17 mmol N m^{-2} d^{-1} and counts refers to the number of depths where that value was estimated. Red bars are export based on satellite data (regional estimate), blue bars are export based on satellite data (global estimate) and black bars are export based on UVP data. Onshore and offshore are combined in panel **b**.

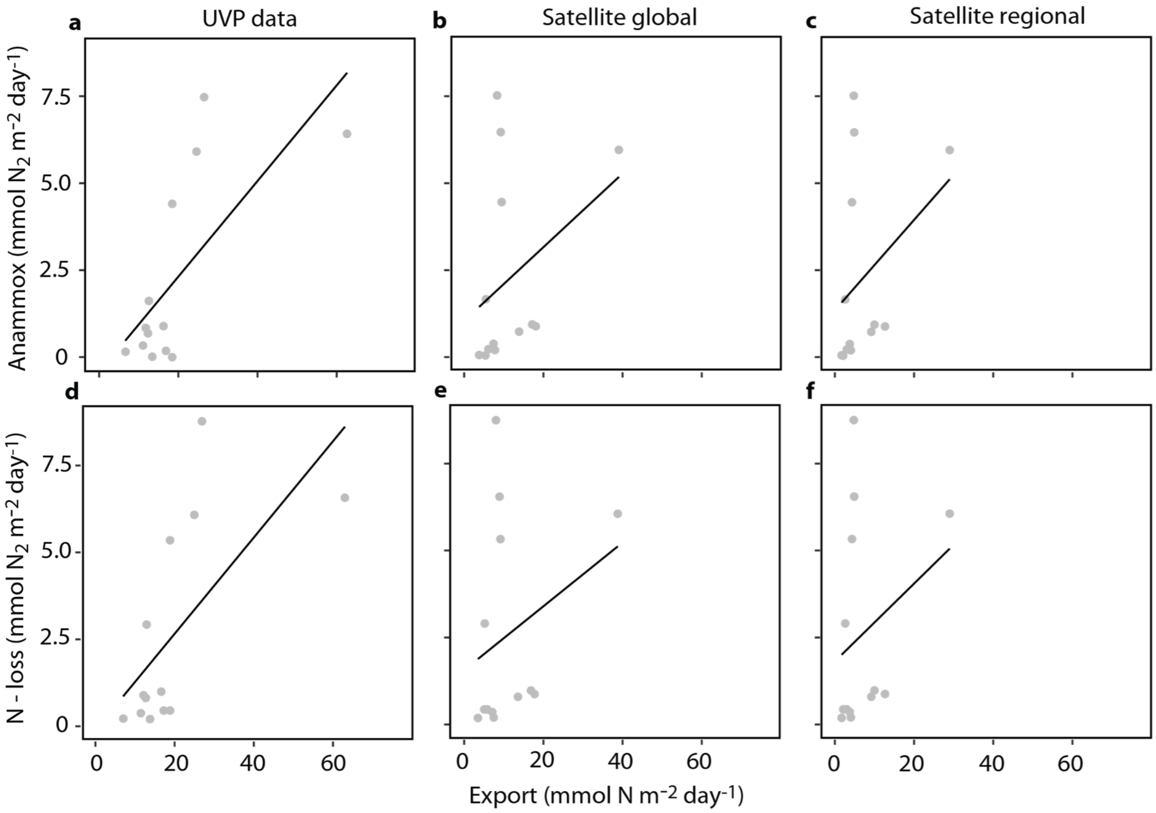

Supplementary Figure 6: Comparison between integrated anammox rates and N-loss rates and the export of organic nitrogen out of the euphotic zone ("export"). Three methods were used to determine export, one based on particle abundances at the base of the euphotic zone from UVP data, measured size specific carbon content of the particles and estimated excess densities[5] (**a**, **c**), and two based on calculating a primary production export-ratio from temperature and chlorophyll concentrations as determined by satellite imagery, either based on a global estimate of export to primary production ratio[4] **(b, e**), or a regional estimate (**c, e**). Integrated anammox rates (in N_2) and shown in the top row, and integrated N loss (i.e. anammox and denitrification) in the second row. Lines indicate the linear regression, for statistics see Supplementary Table 9.

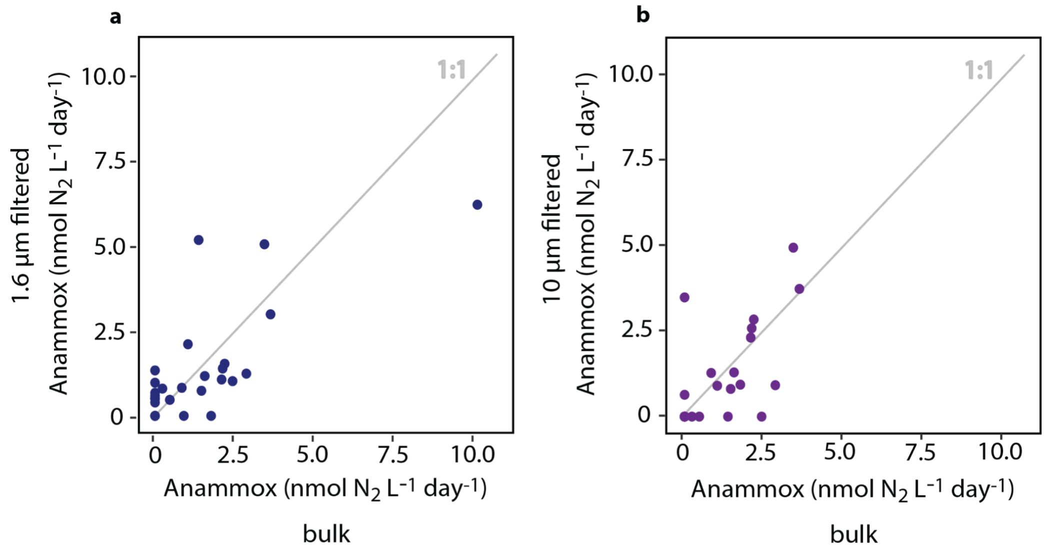

Supplementary Figure 7: Ratio of anammox rates in bulk water with rates in 1.6 µm filtered (panel **a**) and 10 µm filtered water (panel **b**) from the same sampling bottle. As there is no pattern towards increased rates in the bulk water, we assume that anammox cells are free living (see also Figure 3 in the main document), The standard error of the slope for each data point is shown in Supplementary Table 4. 23 samples did not have a significant anammox rate in any of the fractions and are therefore on top of each other at the zero point in this figure.

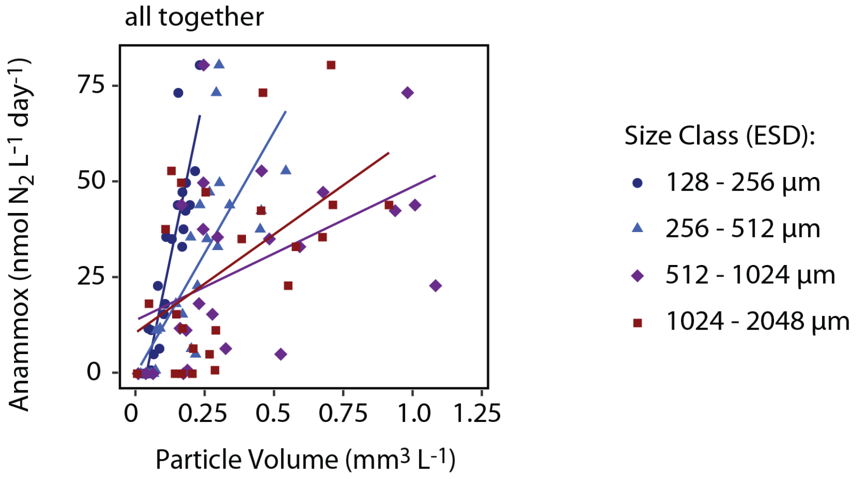

Supplementary Figure 8: Relationship between volumetric anammox rates and particle biovolumes from different size classes in the Peruvian upwelling system during April 2017. Particles throughout the water column were quantified with a UVP and binned into four size classes (each depicted in a different color). Anammox rates were determined from the slope of $^{29}N_2$ production over time in anoxic incubations after addition of $^{15}NO_2^-$ (taking into consideration any contribution to ^{29}N production from denitrification if a denitrification rate with p < 0.05 could be detected). For all particle size classes, there was a significant positive correlation (Spearman's rank correlation; p = < 0.05 between particle biovolume and anammox rates from the anoxic part of the OMZ (O_2 < 1.5 µM *in situ*). The line is the linear regression from which the slope and R^2 was calculated (see Supplementary Table 5 for all relevant statistics). One outlier was removed from the figures and correlation as it was more than 6 SD from the mean in the smallest size fraction and it was the only sample from the euphotic zone (24.3 m depth). Samples from depths where in situ oxygen concentrations were > 1.5 µM were excluded.

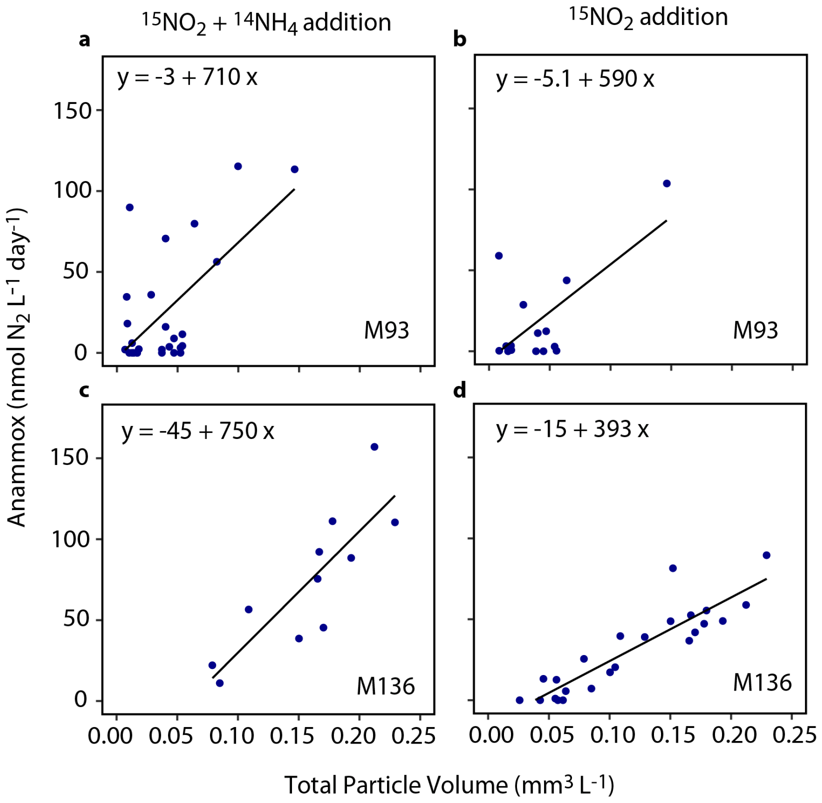

Supplementary Figure 9: Correlations of anammox rates with the abundance of small aggregates in February 2013 (cruise M93, size class 140 - 270 µm, panels **a** and **b**) and in April 2017 (cruise M136, size class 128 - 256 µm, panels **c** and **d**). Anammox rates were determined from $^{15}NO_2$ incubations with an ammonium background (**a** and **c**) and without an ammonium background (**b** and **d**).

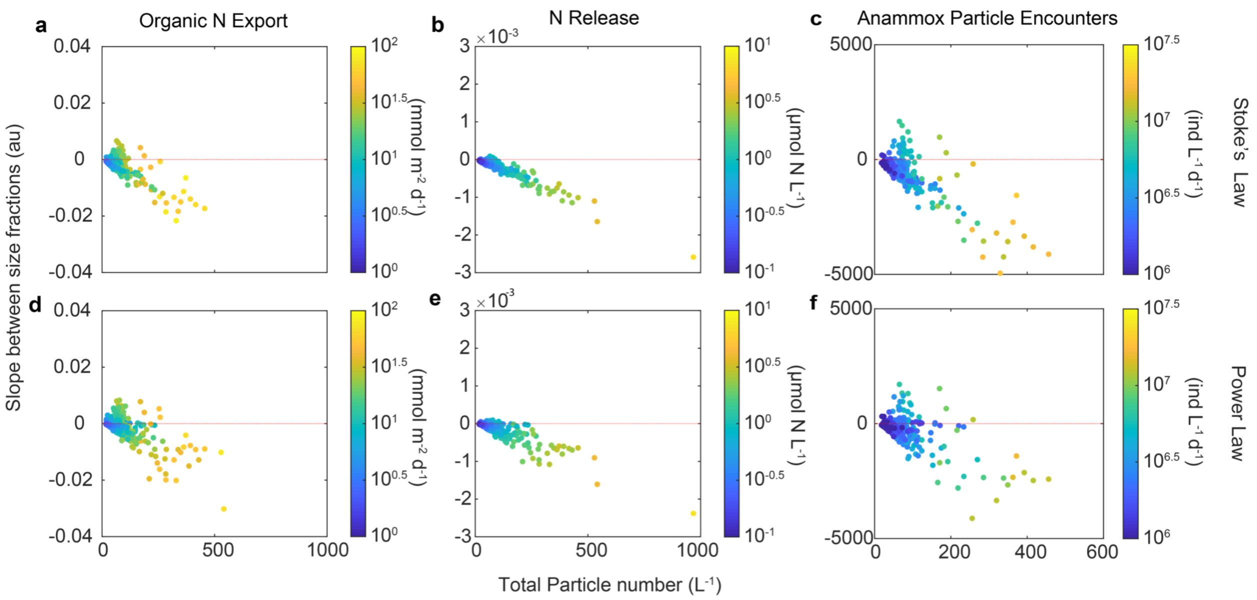

Supplementary Figure 10: Sensitivity of the trend of Organic N Export, N Release and Anammox Particle Encounters along increasing particle sizes based on variations of the particle settling velocity. For each UVP depth, the slope between the four particle sizes was calculated and plotted against the total particle abundance. Slopes below zero indicate that small particles dominate the respective process and - vice versa - slopes above zero indicate that large particles dominate. **a - c** Reference cases based on Stoke's Law with a variable porosity as described in equations (2-5). **d - f** Power law approach for the settling velocity after Kriest[6]: $U = 132r^{0.62}$, which is based on a compilation of various datasets. The results indicate some variability based on the applied relationship, however, overall the results are in the same range and the dominance of small particles for the respective processes persists.

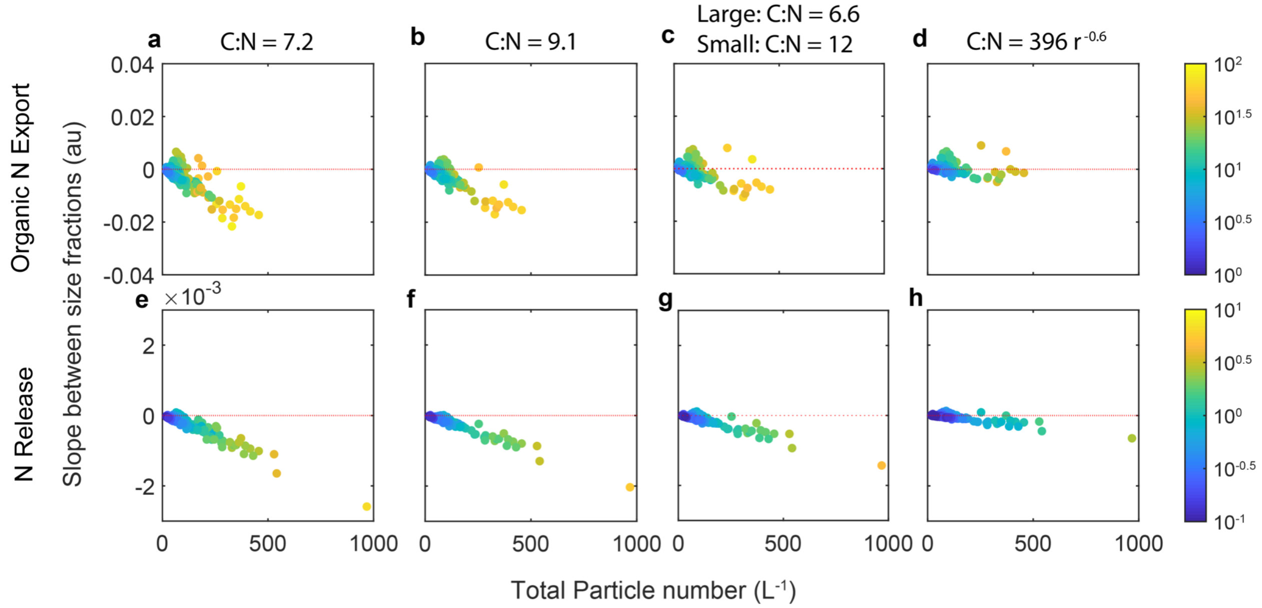

Supplementary Figure 11: Organic N Export and N release for variable C:N ratios.

a+e Reference case for the C:N ratio applied in this study. **b+f** An increased C:N ratio does not change the overall trend but reduces the total organic N export and N release. **c+g** Size-class dependent C:N ratio, where small particles have a C:N ratio of 12 and larger particles a C:N ratio of 6.6. **d+h** Hypothetical scaling relationship for the C:N ratio required to compensate for the effect of small particles. The size dependent C:N ratios are 29, 18, 12, 8 and 5 for 128 µm - 256 µm, 256 µm - 512 µm, 512 µm - 1024 µm and 1024 µm - 2048 µm, respectively.

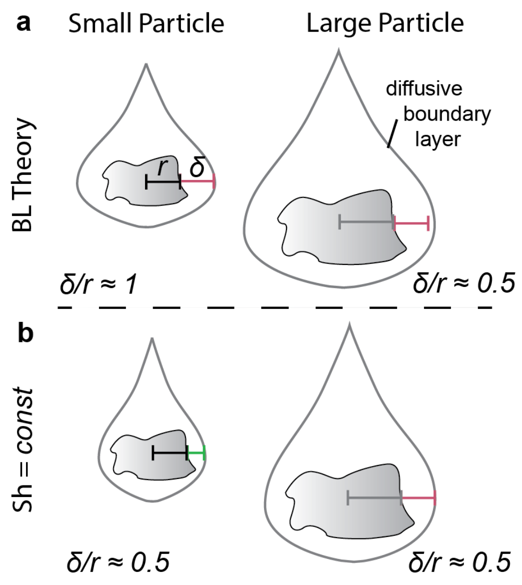

Supplementary Figure 12: Schematic illustration indicating the applied boundary layer theory. The diffusive boundary layer thickness of a settling particle is determined by, both, the ratio of inertial to viscous forces, represented by the Reynolds-number, and the ratio of viscous to diffusive forces, represented by the Schmidt number (see main text). The Reynolds number determines the thickness of the flow boundary layer, while the Schmidt number, determines the amount of solutes diffusing from the particle interstitials into the surrounding seawater. For that reason, the diffusive boundary layer thickness relative to the particle size δ/r depends on both the Reynolds number and Schmidt number, this relationship is represented by the Sherwood (Sh) number (see main text). **a** Applying previously derived scaling laws reveals that for small particles viscous forces are more dominant resulting in a wider DBL relative to the particle size. **b** In case the diffusive boundary layer is hypothesized to be constant relative to the particle size (Sh = *const*), larger particles will have a strongly increased diffusive boundary layer thickness compared to small particles. Please notice that horizontal lines of the same color indicate the same length.

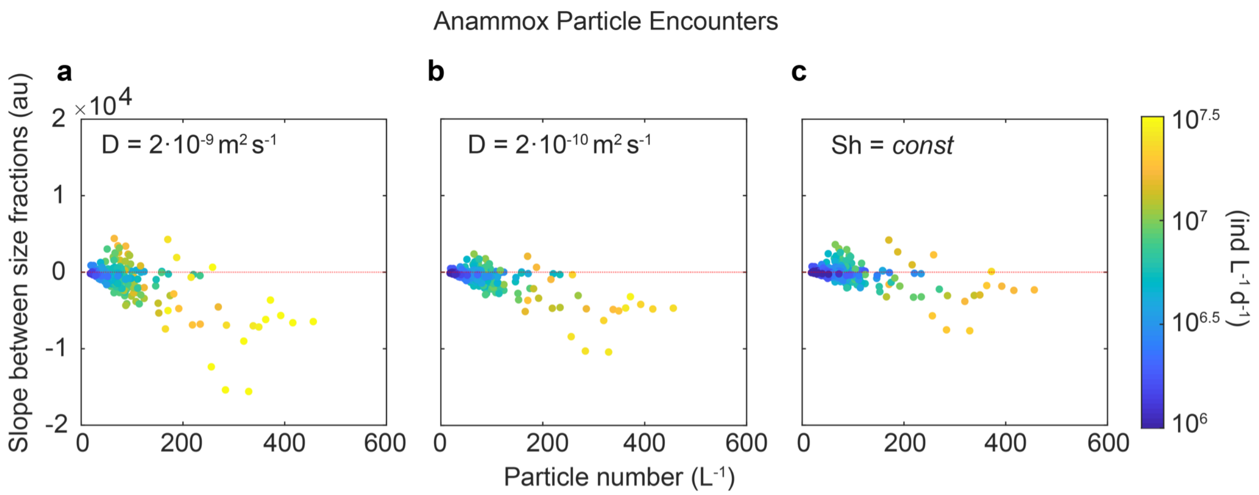

Supplementary Figure 13: Encounter rates of anammox bacteria with the diffusive boundary layer of a sinking particle in response to changing diffusion coefficients. a Assuming a realistic parametrization for the diffusion coefficient, anammox bacteria predominantly encounter small particles, resulting from the large amounts of particles and a larger boundary layer thickness of the particle relative to their size (see also Supplementary Figure 12). **b** The importance of small particles is less prominent with smaller diffusion coefficients or increased viscosities which under in situ conditions could result from colder water temperatures. **c** Assuming a hypothetical, constant ratio of boundary layer thickness and particle size (Sh = *const*), anammox bacteria would roughly encounter as many small particles as they would encounter larger particles.

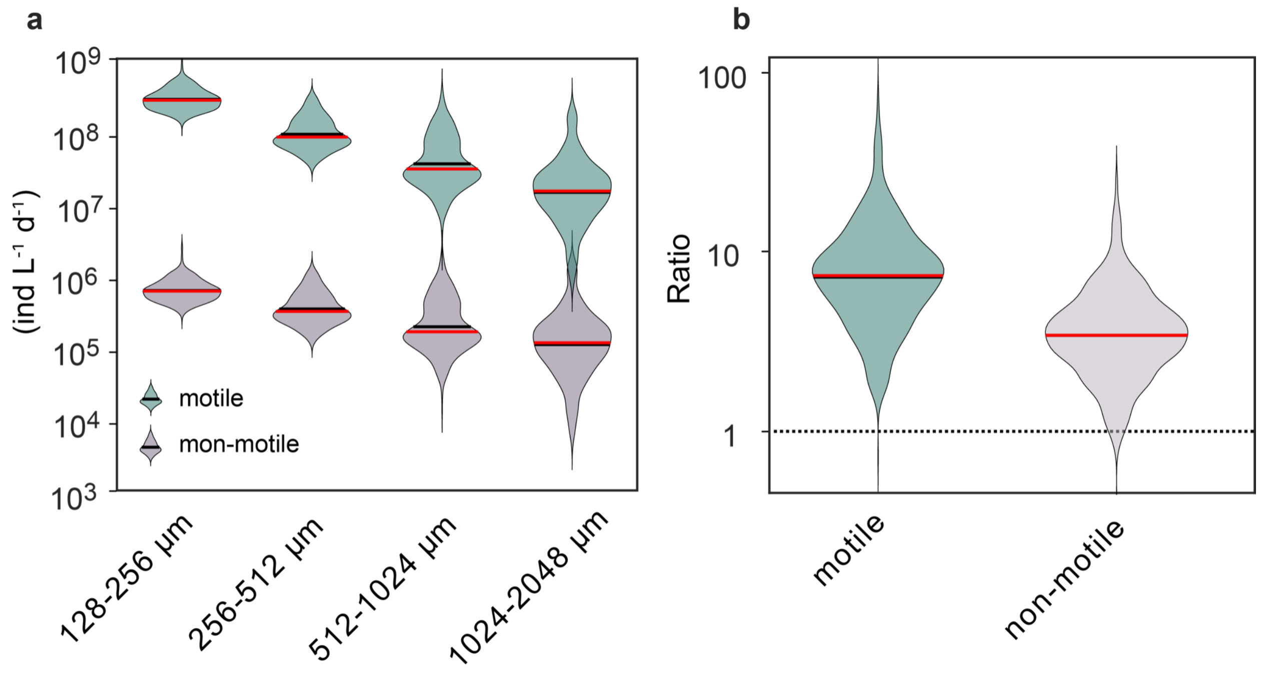

Supplementary Figure 14: Encounter rates of sinking particles with anammox bacteria, assuming motility or non-motility. Within the encounter model, we assume that anammox bacteria are non-motile to estimate a conservative range of encounter rates. However, recently it was shown that encounter rates increase if bacteria are motile[7]. As motility values for anammox bacteria are not described, we adapted a motility model based on an intermediate motility of $1x10^{-9}$ m^2 s^{-1} (ref.[7], eq. 3, green violin plots). Inclusion of motility increases encounter rates by 406 times for smaller particles and 179 times for larger particles. The ratio of encounter with smaller particles compared to encounters with larger particles increased from 2.6 to 3.8. The outlines of the violin plots depict the kernel density estimation of the data points shown. The black line and red line indicate mean and median respectively.

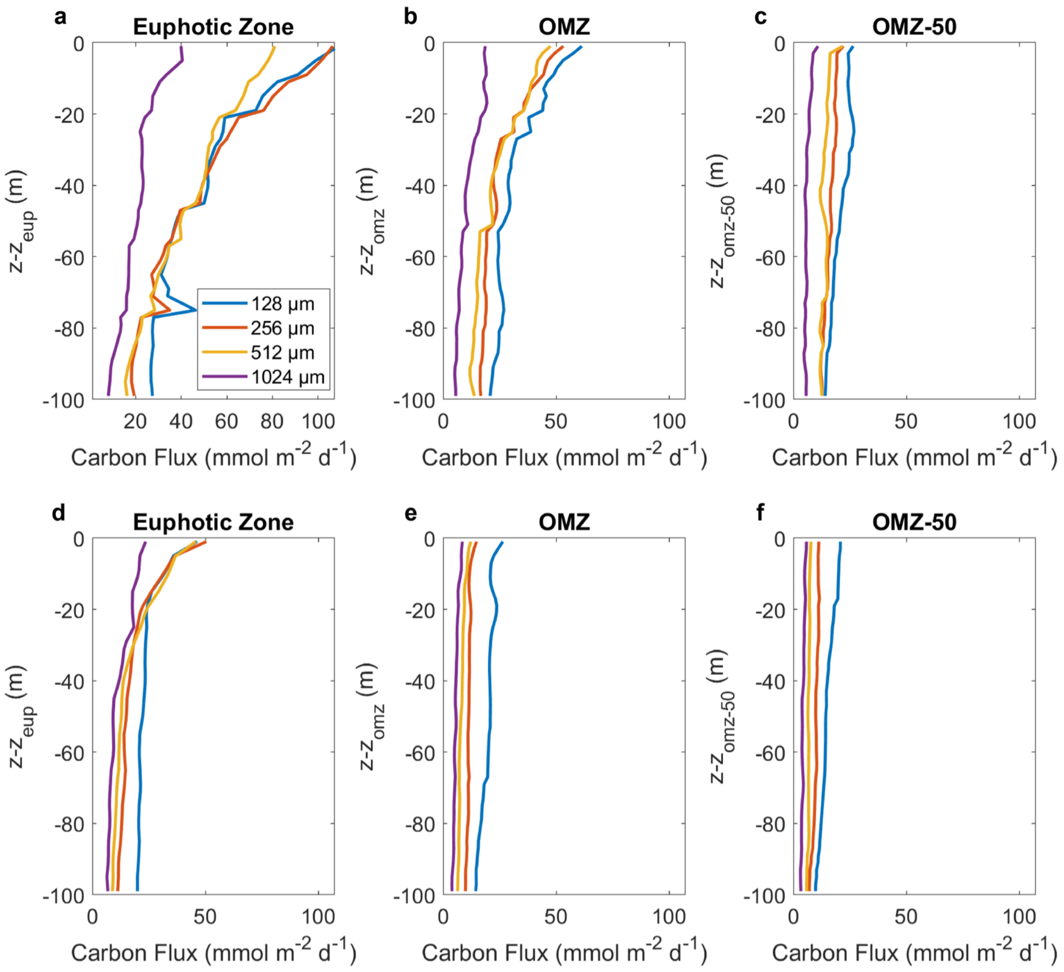

Supplementary Figure 15: Carbon flux (see materials and methods equation 1) for onshore stations **a-c** and offshore stations **d-f**. The average profiles are based on 42 UVP profiles for the onshore stations and 56 UVP profiles for the offshore stations. The profiles were referenced based on the depth of the euphotic zone **a+d**, upper boundary of the OMZ **b+e** and 50 m below the upper boundary of the OMZ **c+f**. We estimated the size specific remineralization length-scale based on the exponential decay of the carbon fluxes (eq. 6). We used three reference depths, namely euphotic depth (z_{eup}), upper boundary of the OMZ (z_{omz}) and the core of the OMZ (z_{omz-50}) which is assumed to start 50 m below the upper boundary of the OMZ. All profiles for the offshore stations (> 600 m water-depth) and onshore stations (< 600 m water-depth) were aligned to the reference depths and subsequently averaged. The information is summarized in Supplementary Table 6.

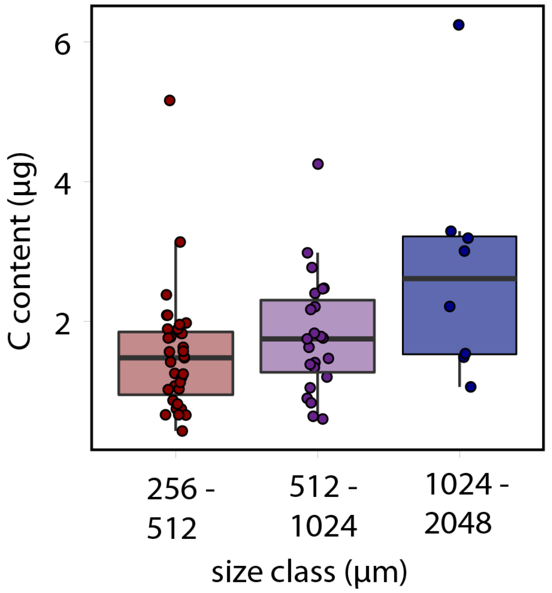

Supplementary Figure 16: Carbon content determined for 66 aggregates sampled using the Marine Snow Catcher. This information was used to rescale the volume-carbon relationship described by Alldredge[8] to match the particles we found in the Peruvian OMZ. Subsequently, the volume-carbon relationship was used to estimate the carbon export for different particle size classes based on their volume (see methods). Boxplots depict the 25–75 % quantile range, with the center line depicting the median (50 % quantile); whiskers encompass data points within 1.5 times the interquartile range.

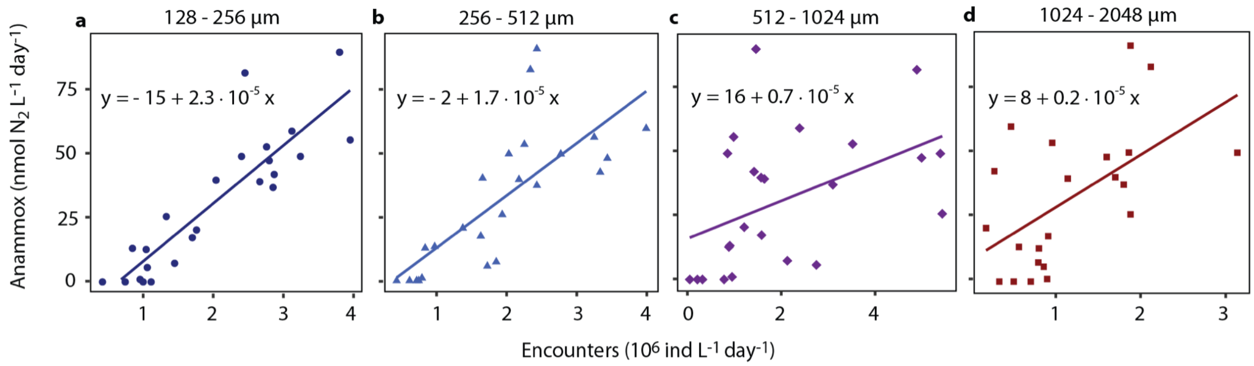

Supplementary Figure 17: Encounter rates of anammox bacteria with particles correlated with anammox rates. Encounter rates were estimated from particle abundance in four different size classes (UVP data) combined with cell numbers determined by Hamersley et al.[9], statistics of the linear regressions can be found in Supplementary Table 10.

Supplementary discussion

Comparison of UVP-based and satellite-based estimates of organic nitrogen export from the euphotic zone

Organic nitrogen export was estimated from UVP measurements based on particle concentrations at the base of the euphotic zone or based on satellite imagery (see methods and Supplementary Figure 5).

UVP-based estimates were highly variable, with a median of 16.6 ± 10.9 mmol N m^{-2} day^{-1} (± is median absolute deviation, see Supplementary Figure 5), which is line with the large variations in particle numbers (Figure 2). The export estimates based on satellite imagery were less variable with a median of 3.7 ± 2.1 mmol N m^{-2} day^{-1}. Satellite-based export production and UVP based organic nitrogen export were significantly correlated (Spearman's rank test, p-value < 2.2 $\cdot 10^{-16}$, slope = 1.1), however median values were significantly different from each other (Wilcoxen signed rank test, p-value < $2.2 \cdot 10^{-16}$). This discrepancy likely results from errors associated with the primary production to export ratio (*pe*-ratio) utilized in the satellite-based estimate. The *pe*-ratio is derived from a previously described relationship between export, chlorophyll concentrations and temperature[4] and is based on an average from a global dataset that does not take the regional and temporal variability of the phytoplankton community and heterotrophic activity into account (see ref.[4]). Therefore, we tested the impact of using a constant *pe*-ratio of 0.4, which is based on measurements in the Peruvian upwelling system[4]. This doubled the estimated median export production of 7.3 ± 3.0 mmol N m^{-2} day^{-1}, highlighting the sensitivity of satellite-based estimates to the poorly constrained *pe*-parameter. The satellite data based approach was also limited by data resolution (spatially: 4 km and temporally: 8 days) and cloud-coverage. These make it difficult to capture the dynamic conditions resulting from mesoscale eddies and lateral intrusions that are present in the Peruvian upwelling system[10-12].

In contrast, the UVP profiles represent a snapshot of the system at the time of sampling, have a higher spatial and temporal resolution and provide detailed information about particle abundances and sizes throughout the water column. Therefore we used the UVP estimates of organic N-export to compare to anammox rates, and to investigate the mechanisms that lead to the relationship between particle volume and anammox rates. It should be noted that for both

satellite and UVP estimates organic N-export was high enough to support the measured anammox rates (Supplementary Figure 6).

Evidence that anammox bacteria are free-living in the bulk water incubations

Bulk water anammox rates were determined from a time series of measurements conducted in 12 mL glass vials that were incubated with $^{15}NO_2^-$ (see methods). Based on the in situ particle abundance determined from the UVP, it is likely that there were between zero and three particles in each of the 12 mL glass vials. If anammox bacteria were directly attached to the particles, then linear rates would not have been expected in the time series. Instead we would expect N_2 production from anammox in some vials and no N_2 production in other vials, rather than a steady increase over time. As we consistently found linear rates of anammox using this approach, despite the likely variation in particle numbers between individual vials, this suggests that anammox rates were not directly affected by the presence or absence of particles. The size fractionation experiments, which showed the same rates in all three treatments (all of which also had linear rates), confirm this result.

Model Parameterization

Carbon specific respiration rates in oxic waters and anoxic waters

Carbon-specific degradation rates were estimated using two different models both of which are based on the carbon flux attenuation calculated from the measured particle profiles (Supplementary Figure 15). The first model represents a simple exponential decay based on a first-order kinetic (equation (6) in methods) and the second model introduces an additional parameter that takes declining particle degradation with depth into account[3], equation (7) in methods). These models are hereafter referred to as the Simple degradation and the Extended degradation model, respectively. Fitting of the model parameters, yielded carbon-specific degradation rates in the upper oxic waters of the stations which were on average 1 d^{-1} (onshore: 0.39 d^{-1}, offshore: 1.6 d^{-1}) and 0.5 d^{-1} (onshore: 0.46 d^{-1}, offshore: 0.54 d^{-1}) using the Simple and Extended degradation model, respectively.

While sinking, particles are remineralized, but also undergo aggregation and fragmentation, which can impact carbon specific degradation rates when they are calculated from particle

profiles. Fragmentation and disaggregation is driven by zooplankton feeding, microbial degradation and shear in the turbulent water column[13-15]. In effect this leads to the “disappearance” of larger particles in profiles (potentially leading to an overestimation of degradation rates in the larger size classes) and results in the formation of smaller particles masking remineralization (potentially leading to an underestimation of degradation in the smaller size classes). The difference in degradation rate estimated for the offshore stations by the two models might indicate that fragmentation was playing a role in the upper water column which declined with depth. This is more likely to be captured in the Extended degradation model as it estimates the overall shape of the flux attenuation profiles better (see methods, eq. 7). Furthermore, in the extended model, the higher degradation rates of the larger particles (on average 0.65 d^{-1}, offshore: 0.58 d^{-1}, onshore: 0.71 d^{-1}) compared to the smaller particles 0.35 d^{-1} (offshore: 0.33 d^{-1}, onshore: 0.36 d^{-1}), might also indicate that fragmentation was occurring in the upper oxic waters.

In order to investigate the possible impact of fragmentation in the upper oxic waters, we calculated the carbon-specific degradation rate based on the integration of the entire size spectrum using the Extended model (in contrast to using the profiles from the size classes separately, as above). This approach largely excludes shifts from the larger to the smaller size classes due to fragmentation. In this case, carbon-specific degradation was estimated to be 0.48 d^{-1} (offshore: 0.45 d^{-1}, onshore: 0.52 d^{-1}) in the upper oxic water column. The difference between the fragmentation corrected degradation rate and the rates presented above indicates that the degradation of larger particles might be overestimated by 33 % (onshore: 29 %, offshore: 37 %) while the degradation of smaller particles is underestimated by 27 % (onshore: 17 %, offshore: 31 %).

The same approach was used to estimate carbon specific degradation rates below the oxic-anoxic interface. Fitting of the model parameters yielded carbon-specific respiration rates which were on average 0.19 d^{-1} (onshore: 0.22 d^{-1}, offshore: 0.15 d^{-1}) and 0.3 d^{-1} (onshore: 0.2 d^{-1} offshore: 0.44 d^{-1}) using the Simple and Extended degradation model, respectively. Using the extended model, the degradation associated with larger particles was higher on average (0.42 d^{-1}; onshore: 0.3 d^{-1}, offshore: 0.55 d^{-1}) than the degradation associated with smaller particles (0.22 d^{-1}; onshore: 0.1 d^{-1} offshore: 0.34 d^{-1}). When the whole particle size spectrum was integrated the average carbon-specific degradation rate within the anoxic waters was estimated to be 0.29 d^{-1} (onshore: 0.12 d^{-1}, offshore: 0.46 d^{-1}). This indicates that the degradation of larger particles might be overestimated by 85 % (onshore: 150 %, offshore: 20 %) and that of smaller

particles might be underestimated by 22 % (onshore 17 %, offshore 26 %). All of these approaches are estimates of carbon specific degradation, as necessarily they cannot represent all of the complex processes that lead to carbon flux attenuation in situ. However, they imply that the initial applications of the simple and extended model are conservative in regards to the trend between smaller and larger particles, and that smaller particles play an even more important role relatively than larger particles in terms of carbon specific degradation and therefore N-release. All of the presented estimates are in line with previous carbon-specific respiration rates from the Eastern Tropical North Pacific determined from measurements of oxygen respiration (0.13 - 5 d^{-1}, ref.[2]).

Organic nitrogen export into the OMZ and remineralization

We tested how varying the parameters in the model impacted the estimates of organic nitrogen export into the OMZ and remineralization of organic matter within the OMZ (equations (1-4) in the methods). The major assumptions included in these models used to make these estimates are the particle settling velocity, carbon specific degradation rate (see above), and the conversion factor that relates carbon to nitrogen export (i.e. the C:N ratio).

We compared two widely applied methods to determine settling velocity based on particle size. The first equation (equations (2-5) in the methods) is based on Stoke's Law with a size-dependent porosity while the second equation uses a general power law approach[6]: $U= 132r^{0.62}$, which is based on a compilation of various datasets (hereafter referred to as the Stoke's and Power Law settling velocity approach, respectively). The two approaches gave similar results for both N-export and remineralization (Supplementary Figure 10 a and d), however, both N-export and remineralization were lower using the Power law approach and the magnitude was decreased by 43 %, 32 %, 22 %, and 9 % percent in the four different size classes, respectively. The comparably higher export of organic N associated with smaller particles persisted using the power law approach but the ratio associated with smaller vs larger particles changed from to 1.2. The remineralization (or N release) ratio between the smaller to larger particles changed from 4.6 to 3.7.

We assumed a C:N ratio of 7.2 based on previous measurements from the same region[16]. However, preferential N-remineralization seems to occur in sinking organic matter[17,18]. To test the effect that this would have on organic N-export estimates, we first changed the C:N

ratio in the model to 9.1 (Supplementary Figure 11 panels b and f) based on measurements from deep sediment traps near Peru[18]. The increased C:N ratio decreased the organic N-export by 21 % for each of the four different size classes but did not affect the ratio between organic N-export by smaller and larger particles.

There is also ample evidence in the literature that the preferential remineralization and uptake of N that occurs during heterotrophic organic matter degradation leads to increasing C:N ratios as material ages[19-21]. Therefore, theoretically, preferential N-remineralization over time could lead to the smaller, slower sinking and older particles having a higher C:N ratio than that of larger, faster sinking, younger particles. It should be noted however, that most of these studies focus on the transformation of particulate to dissolved organic matter[20,22], or ageing within the dissolved organic matter pool[19,21], and that differences in C:N ratio between particle size classes are poorly constrained. However, to investigate the impact of such an effect we altered the C:N ratios in our model for the individual size classes (to 6.6 for the larger particles and 12 for the smaller particles; Supplementary Figure 11 panels c and g). This reduces the organic N-export by the smaller particles by 68 % and increases it by 7 % for the larger size classes. Based on this extreme case, the ratio of organic N-export between the smaller to larger particles changed from 3.4 to 1.9. In fact, in the model a hypothetical, size-dependent C:N scaling relationship of 396 $r^{-0.61}$ would be needed to compensate for the trend between the particle size fractions. In this case, the smallest particle size class would have a 5.8 times higher C:N ratio than the largest size class.

Concerning the N-release estimates - if preferential N-remineralization of the organic matter is taking place while particles sink, then this implies that our N-release rates are underestimated. Preferential N-remineralization and the associated enhanced release of N into the water column would lead to a C:N ratio that gradually increases with depth. Therefore, the C and N mass fluxes would be decoupled leading to element specific degradation rates. Implementation of C and N specific degradation rates within the models would require accurate measurements of C:N ratios through-out the water column and for different particle types.

Encounters between anammox bacteria and particles

Encounter rates were calculated based on particle size, abundance, settling velocity and boundary layer theory. We also assume that anammox bacteria are passive tracers in the water

column (i.e non-motile) and that the bacteria only need to be near the boundary layer of a particle to utilize the released N. This differs from previous literature which has mainly focused on the colonization of particles by motile bacteria (e.g. refs.[7,23]). The boundary layer associated with particles of different size classes and particle settling velocities are the key parameters determining the results from the model.

The changes in the flow-regime lead to large variability in the boundary layer thicknesses of small and large particles. Smaller and slower sinking particles have a wide boundary layer thickness relative to their size compared to larger and faster settling particles (Supplementary Figure 12). Therefore, anammox bacteria encountered the boundary layer of small particles 2.6 times more often. When we applied a different size-sinking velocity relationship (see above), the trend remains similar but anammox bacteria encounter small particles 1.5 times more often (Supplementary Figure 10). In case the boundary layer thickness would, hypothetically, be assumed to be constant relative to their size, anammox bacteria would encounter as many small particles as they encounter large particles (Supplementary Figure 13 c).

We also tested the impact of assuming that anammox bacteria are non-motile, using a particle encounter model developed for motile bacteria[7]. In comparison to the non-motile model, encounter rates within each size class increased by 2 to almost 3 orders of magnitude (Supplementary Figure 14). Encounter rates between anammox bacteria and smaller particles showed a stronger increase compared to those between anammox bacteria and larger particles, increasing the ratio from 2.6 (as found in the first iteration of the non-motile model; see above) to 3.8.

The influence of suspended particles

Suspended particles, which we assume are not imaged by the UVP and are therefore not included in the models discussed above, might have an additional influence on N-loss in the OMZ that is not addressed by our approach. Suspended particles can be present in the water column through 1) spontaneous assembly of free polymer chains into microgels (see ref.[24]), however such transparent gels are formed of material which likely has low nitrogen content[24]) and as such would contribute little to organic nitrogen release 2) through fragmentation or degradation of sinking particles to the extent that they stop sinking. In the second case, assuming steady state conditions, the presence of suspended particles is unlikely to have had a large impact on our organic N-export rates. This is supported by the similar export estimates we

found using a UVP independent approach (see above) and the similarity of our fluxes to those in Cavan et al.[2]. The influence of suspended particles on N-release and encounter rates is harder to predict, as it requires a better understanding of the distribution of suspended particles, their nitrogen contents and their remineralization rates. However, it seems likely that remineralization would continue in very small particles that have become suspended. As the current approach does not take degradation and release of nitrogen from suspended particles into account it is likely that our modelled estimates are conservative.

Bibliography

1. Picheral, M., Colin, S. & Irisson, J. O. EcoTaxa, a tool for the taxonomic classification of images. *URL: http://ecotaxa. obs-vlfr.fr* (2017).
2. Cavan, E. L., Trimmer, M., Shelley, F. & Sanders, R. Remineralization of particulate organic carbon in an ocean oxygen minimum zone. *Nat. Commun.* **8**, 14847 (2017). https://doi.org/10.1038/ncomms14847
3. De Soto, F., Ceballos-Romero, E. & Villa-Alfageme, M. A microscopic simulation of particle flux in ocean waters: Application to radioactive pair disequilibrium. *Geochim. Cosmochim. Acta* **239**, 136–158 (2018). https://doi.org/10.1016/j.gca.2018.07.031
4. Dunne, J. P., Armstrong, R. A., Gnanadesikan, A. & Sarmiento, J. L. Empirical and mechanistic models for the particle export ratio. *Global Biogeochem. Cycles* **19**, (2005). https://doi.org/10.1029/2004GB002390
5. Alldredge, A. L. & Gotschalk, C. In situ settling behavior of marine snow. *Limnol. Oceanogr.* **33**, 339–351 (1988). https://doi.org/10.4319/lo.1988.33.3.0339
6. Kriest, I. Different parameterizations of marine snow in a 1D-model and their influence on representation of marine snow, nitrogen budget and sedimentation. *Deep Sea Research Part I: Oceanographic Research Papers* **49**, 2133–2162 (2002). https://doi.org/10.1016/S0967-0637(02)00127-9
7. Lambert, B. S., Fernandez, V. I. & Stocker, R. Motility drives bacterial encounter with particles responsible for carbon export throughout the ocean. *Limnol. Oceanogr.* **4**, 113–118 (2019). https://doi.org/10.1002/lol2.10113
8. Alldredge, A. The carbon, nitrogen and mass content of marine snow as a function of aggregate size. *Deep Sea Research Part I: Oceanographic Research Papers* **45**, 529–541 (1998). https://doi.org/10.1016/S0967-0637(97)00048-4
9. Hamersley, M. R. *et al.* Anaerobic ammonium oxidation in the Peruvian oxygen minimum zone. *Limnol. Oceanogr.* **52**, 923–933 (2007). https://doi.org/10.4319/lo.2007.52.3.0923
10. Callbeck, C. M., Lavik, G., Stramma, L., Kuypers, M. M. M. & Bristow, L. A. Enhanced Nitrogen Loss by Eddy-Induced Vertical Transport in the Offshore Peruvian Oxygen Minimum Zone. *PLoS ONE* **12**, e0170059 (2017). https://doi.org/10.1371/journal.pone.0170059
11. Thomsen, S. *et al.* The formation of a subsurface anticyclonic eddy in the Peru-Chile Undercurrent and its impact on the near-coastal salinity, oxygen, and nutrient distributions. *J. Geophys. Res. Oceans* **121**, 476–501 (2016). https://doi.org/10.1002/2015JC010878
12. Thomsen, S. *et al.* Do submesoscale frontal processes ventilate the oxygen minimum zone off Peru? *Geophys. Res. Lett.* **43**, 8133–8142 (2016). https://doi.org/10.1002/2016GL070548
13. Briggs, N., Dall'Olmo, G. & Claustre, H. Major role of particle fragmentation in regulating biological sequestration of CO2 by the oceans. *Science* **367**, 791–793 (2020). https://doi.org/10.1126/science.aay1790
14. Lampitt, R. S., Noji, T. & von Bodungen, B. What happens to zooplankton faecal pellets? Implications for material flux. *Mar. Biol.* **104**, 15–23 (1990). https://doi.org/10.1007/BF01313152

15. Mayor, D. J., Sanders, R., Giering, S. L. C. & Anderson, T. R. Microbial gardening in the ocean's twilight zone: detritivorous metazoans benefit from fragmenting, rather than ingesting, sinking detritus: fragmentation of refractory detritus by zooplankton beneath the euphotic zone stimulates the harvestable production of labile and nutritious microbial biomass. *Bioessays* **36**, 1132–1137 (2014). https://doi.org/10.1002/bies.201400100
16. Kalvelage, T. *et al.* Nitrogen cycling driven by organic matter export in the South Pacific oxygen minimum zone. *Nature Geosci.* **6**, 228–234 (2013). https://doi.org/10.1038/ngeo1739
17. Van Mooy, B. A. ., Keil, R. G. & Devol, A. H. Impact of suboxia on sinking particulate organic carbon: Enhanced carbon flux and preferential degradation of amino acids via denitrification. *Geochim. Cosmochim. Acta* **66**, 457–465 (2002). https://doi.org/10.1016/S0016-7037(01)00787-6
18. Martin, J. H., Knauer, G. A., Karl, D. M. & Broenkow, W. W. VERTEX: carbon cycling in the northeast Pacific. *Deep Sea Research Part A. Oceanographic Research Papers* **34**, 267–285 (1987). https://doi.org/10.1016/0198-0149(87)90086-0
19. Hach, P. F. *et al.* Rapid microbial diversification of dissolved organic matter in oceanic surface waters leads to carbon sequestration. *Sci. Rep.* **10**, 13025 (2020). https://doi.org/10.1038/s41598-020-69930-y
20. Benner, R. & Amon, R. M. W. The size-reactivity continuum of major bioelements in the ocean. *Ann. Rev. Mar. Sci.* **7**, 185–205 (2015). https://doi.org/10.1146/annurev-marine-010213-135126
21. Walker, B. D., Beaupré, S. R., Guilderson, T. P., McCarthy, M. D. & Druffel, E. R. M. Pacific carbon cycling constrained by organic matter size, age and composition relationships. *Nature Geosci.* **9**, 888–891 (2016). https://doi.org/10.1038/ngeo2830
22. Druffel, E. R. M. & Williams, P. M. Identification of a deep marine source of particulate organic carbon using bomb 14C. *Nature* **347**, 172–174 (1990). https://doi.org/10.1038/347172a0
23. Kiørboe, T. & Jackson, G. A. Marine snow, organic solute plumes, and optimal chemosensory behavior of bacteria. *Limnol. Oceanogr.* **46**, 1309–1318 (2001). https://doi.org/10.4319/lo.2001.46.6.1309
24. Engel, A., Endres, S., Galgani, L. & Schartau, M. Marvelous Marine Microgels: On the Distribution and Impact of Gel-Like Particles in the Oceanic Water-Column. *Front. Mar. Sci.* **7**, (2020). https://doi.org/10.3389/fmars.2020.00405
