# Supplementary figures and images for: Small sinking particles control anammox rates in the Peruvian oxygen minimum zone
Source: Nat Commun. 2021 May 28;12:3235. doi: 10.1038/s41467-021-23340-4 (PMC8163745; doi:10.1038/s41467-021-23340-4)

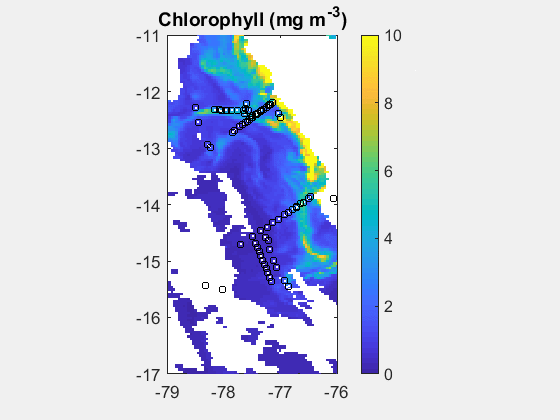

Supplement: Supplementary file 2 — Supplementary Video [file 41467_2021_23340_MOESM2_ESM.gif]
