# Supplementary material for: Small sinking particles control anammox rates in the Peruvian oxygen minimum zone
Source: Nat Commun. 2021 May 28;12:3235. doi: 10.1038/s41467-021-23340-4 (PMC8163745; doi:10.1038/s41467-021-23340-4)
Supplement: Supplementary file 4 — Description of Additional Supplementary Files [file 41467_2021_23340_MOESM4_ESM.docx]

Description of additional supplementary files

Title: Supplementary Video

Description: Chlorophyll concentrations and variability during April 2017 (cruise M136). Chlorophyll concentrations are based on satellite data and were taken from Giovanni, NASA (https://giovanni.gsfc.nasa.gov/giovanni/, last visit: 5/11/2020). Stations at which CTD casts were taken during that time period are marked with circles in the map (Filename: Chl_a_M136.gif).

Title: Supplementary Table 1

Description: List of all incubated samples with their temporal and spatial information, physico-chemical data, N loss rates and particle abundances. a Incubation of unfiltered samples (Tab labelled Table_1a_bulk in excel file) b Incubation of size fractionated samples (Tab labelled Table_1b_size_fractionation in excel file) c Comparison of incubations from April 2017 (Cruise M136) with samples from February 2013 (Cruise M93, tab labelled Table_1c_M93_comparison in excel file).

Title: Supplementary Table 2

Description: List of all sampled stations with their temporal and spatial information, OMZ extent, chlorophyll a content, anammox encounter rates, particle export and resulting N release and N flux and i
